# Modulation of the gut microbiota and the microbial-produced gut metabolites by diclofenac exposure and selenium supplementation

**DOI:** 10.1007/s11356-025-36233-6

**Published:** 2025-03-18

**Authors:** Gema Rodríguez-Moro, Raúl Cabrera-Rubio, Marta Selma-Royo, José Antonio Gómez-Morlote, Maria Carmen Collado, Nieves Abril, Tamara García-Barrera

**Affiliations:** 1 Department of Chemistry, Faculty of Experimental Sciences, Research Center of Natural Resources, Health and the Environment (RENSMA), University of Huelva, Fuerzas Armadas Ave, 21007 Huelva, Spain; 2 Department of Biotechnology, Institute of Agrochemistry and Food Technology-National Research Council (IATA-CSIC), Agustin Escardino 7, 46980 Paterna, Valencia Spain; 3 Department of Biochemistry and Molecular Biology, University of Córdoba, Campus de Rabanales, Edificio Severo Ochoa, 14071 Córdoba, Spain

**Keywords:** Diclofenac, Selenium, Gut metabolomics, Gut microbiota, Functional food, Mass spectrometry

## Abstract

Diclofenac (DCF) exposure is of great concern due to the ecotoxicological risk linked with a decline of vulture populations in Southeast Asia, but also because it can affect the reproduction and neurotoxicity in mammals. Otherwise, selenium (Se) is an antioxidant essential element with key roles in health and with antagonistic action against pollutants, but in some cases with a synergistic effect. To investigate the potential intertwined mechanisms between DCF, Se, and gut microbiota, gut metabolomic and gut microbiota profiles were determined in mice after DCF exposure and Se supplementation. Speciation of selenoproteins in plasma was carried out by isotopic dilution analysis to quantify the levels of selenoproteins. Significant differences in the levels of 79% of the gut metabolites were determined after DCF exposure. The most significant altered pathway in DCF and DCF-Se groups is the primary bile biosynthesis, being the only pathway altered in mice exposed to DCF, while in DCF-Se, the metabolism of galactose and linoleic acid is also altered. Moreover, specific associations between specific gut microbiota and metabolites were determined in the studied mice groups suggesting intertwined mechanisms. Selenium supplementation modulated the gut metabolic and microbiota profiles affected by DCF.

**Supplementary Information:**

The online version contains supplementary material available at 10.1007/s11356-025-36233-6.

## Introduction

The ecological risk related to the presence of pharmaceutically active compounds (PACs) in the environment is of growing interest due to their impact on ecosystems and the neurotoxic effects observed in mammals and aquatic organisms derived from exposure to PACs such as DCF and its metabolites (Aygün et al. 2012). DCF is a non-steroidal anti-inflammatory drug (NSAID) used to treat injured cattle that caused near the extinction of vultures (decline of 99%) on the Asian subcontinent due to its presence in food (Oaks et al. 2004). In Europe, the veterinary use of DCF is greatly restricted, but currently allowed by both the European Union and the national governments of Spain and Italy. Moreover, the use of DCF in humans could pollute the surface water that finally reach and overload the treatment plants constituting a risk for drinking water consumption (Lonappan et al. 2016). Nowadays, the doses of NSAIDs consumed by humans for therapeutic purpose are considered safe and no visible effects on human health yet, but recently there is growing concern about future problems related to chronic sources of exposure, as its presence in edible plants (Klampfl 2019). Among the future effects of NSAIDs, including DCF, on human health are hepatotoxicity, genotoxicity, DNA damage, stomach ulcers, respiratory problems, chronic depression, or congenital problems (Rastogi et al. 2021).

Accumulated evidence suggests also a critical role of gut microbiota in the biotransformation and bioavailability of xenobiotics such as PACs including DCF (Ahlawat et al. 2021). Interestingly, there are previous studies that demonstrated a protective effect of selenium (Se) on DCF-induced reproductive toxicity in mammals (Owumi et al. 2020). Selenium is an essential trace element well-known for its antagonistic action against other pollutants (García-Barrera et al. 2012) that can also impact on the gut microbiota increasing some health-relevant taxa (e.g., *Lactobacillus*) (Callejón-Leblic et al. 2021), gut metabolites (Callejón-Leblic et al. 2022), and brain metabolites.

The importance of the gut-brain crosstalk through the known gut-brain axis has been widely reported (Morais et al. 2021) as well as the neurotoxic damage of DCF (Sathishkumar et al. 2021) and the impact of DCF exposure on gut microbiota (Zádori et al. 2023). However, the impact on the gut metabolome and the potential association with gut microbes have not been deeply studied, especially in Se-supplemented mammals.

In this work, we described the impact of DCF exposure and Se supplementation in the plasma selenoproteome, gut microbiota, and gut metabolites as well as the association of metabolites with specific microbial traits in order to ascertain the host-microbe interactions.

## Experimental section

### Experimental design with animals and dosage information

A total of 30 *Mus musculus* male (inbred BALB/c) mice of 8 weeks of age (23–25 g) were obtained from Charles River Laboratories and acclimated for 3 days with free access to food and water at room temperature and a cycle of 12 h light/dark. After the acclimatation period, mice were randomly divided into three groups (ten mice per group): (a) control group (C), (b) diclofenac diet (DCF), with a dose of 20 mg/kg of chow, and (c) diet that combines the doses of sodium selenite (with a dose of 0.65 mg/kg of chow) and diclofenac (at the same doses that group DCF). The sodium selenite dose was three times the usual selenium ratio in a diet for rodents, considered non-toxic under our experimental conditions and accords to previous studies (Amato et al. 2020; Zarrinpar et al. 2018).

The DCF dose was selected based on environmentally relevant concentrations, considering DCF’s potential for bioaccumulation and biomagnification along the trophic chain (Peters et al. 2022). Additionally, we took into account typical doses employed in murine experiments reported in the literature (e.g., Mayorek et al. (2010); Selvaraj et al. (2023); Thai et al. (2023)), as well as theLD50 for DCF established at 95–130 mg /kg per day (Gomaa 2018; He et al. 2019). We preferred a continuous exposure instead of an acute one to simulate conditions of accidental exposure to DCF present in the environment. In order to simulate the conditions of accidental and prolonged exposure to DCF present in the water, we opted for a continuous exposure rather than an acute one. After 14 days of continuous DCF exposure, the mice were exsanguinated by cardiac puncture and dissected using a ceramic scalpel. The large intestine contents from cecum and colon were collected from each mouse and stored at − 80 °C until the analysis.

### Speciation of selenoproteins in mice plasma

#### Sample preparation

Plasma samples were filtered using PVDF (polyvinylidene difluoride) filters of 20 mm diameter and 0.45 µm of pore size in order to avoid overloading the columns chromatographic.

#### SEC-AF-SUID-ICP-QQQ-MS experimental conditions

Quantification of selenoproteins in plasma from mice *Mus musculus* was carried out by high perform liquid chromatography (HPLC) coupled to an inductively coupled plasma mass spectrometer (ICP-MS) following the previous methodology described by other authors (Callejón-Leblic et al. 2021). See supporting information for more details.

### Untargeted gut metabolomic analysis combining GC–MS and UPLC-QTOF-MS

#### Sample treatment

Firstly, gut contents (cecum and colon) were lyophilized and after homogenization with a vortex. To this end, 10 mg of dried sample was taken and dissolved with 300 µL of a cold mixture containing 80:20 (v/v) methanol (MeOH) and methyl tert-butyl ether (MTBE) for metabolites extraction. The mixture was homogenized for 30 min with a cell disruption pellet mixer and then centrifuged at 12,825 g for 10 min at 4 °C. An aliquot of 50 µL of the supernatant was taken for GC–MS analysis and 200 µL for UPLC-QTOF-MS analysis in two ionization modes, positive and negative. Both extracts were dried using a SpeedVac system.

For GC–MS analysis, samples were derivatized in two steps with derivatizing reagents (Rey-Stolle et al. 2022). First, dried extracts were added 50 µL of 15 mg/mL methoxyamine in piridine to methoximation process. The vials were then closed and vortexed for 15 min. Subsequently, the samples were covered with aluminum foil and incubated at room temperature and in darkness condition for 16 h. As a second step, a silylation process was carried out by adding 50 µL of *N*-methyl-*N*-(trimethylsilyl)trifluoroacetamide (MSTFA). The resulting mixture was vortexed and incubated at 70 °C for 1 h. Finally, the supernatant was picked up for further analysis.

For UPLC-MS, the resulting dried extracts were redissolved in 50 µL of MeOH:MTBE 80:20 (v/v) and transferred to vials for the analysis.

In addition, to check the stability of the analytical signal, five quality control (QC) samples were analyzed during the whole process. QCs were prepared by adding the same amount of all the samples studied and were treated with the same procedure described above.

#### GC–MS experimental conditions

The experimental parameters for metabolomics using GC–MS are described in the Supporting information.

#### UPLC-QTOF-MS conditions

In order to increase the metabolite coverage, GC–MS was combined with UHPLC-QTOF-MS. The experimental parameters for metabolomics using UHPLC-QTOF-MS are described in the Supporting information.

#### Data processing

Data processing after GC–MS analysis was carried out with the free access XCMS software on the R platform. Firstly, the files were converted into CDF format and then, they were processed following the methodology described by Katajamaa and Oresic (2007) using XCMS-optimized parameters, to extract the maximum possible information, such as filtering, feature detection peak alignment, and data normalization.

After UPLC-QTOF-MS analysis, raw data processing was performed with the Mass Profinder® software version B.10.00 (Agilent Technologies, Waldbronn, Germany). This program consists of different Molecular Feature Extraction (MFE) algorithms, which are used to perform the necessary processing steps, including peak selection, cleaning the background, and alignment of peak. Once all the information is obtained, the data is transferred to Mass Profiler® Professional (MPP) B.8.00 to carry out an univariate and multivariate analysis.

#### Statistical analysis

Data obtained by both analysis techniques were subjected to univariate and multivariate analysis to determine if the studied groups are statistically different and to identify the gut metabolites altered after exposure to DCF and supplementation with Se.

For GC–MS data, the SIMCA-P software (version 11.5, UMetrics, Umea, Sweden) was used, while data obtained by UPLC-QTOF-MS were statistically analyzed by the software Mass Profiler Professional (MPP) from Agilent Technologies. In both cases, multivariate analyses were developed to compare the gut metabolomic profiles and to determine the variables (metabolites), with VIP > 1 considered significant for each comparison. The quality of the models was evaluated with the parameters shown in Table S1. Later, after annotation of metabolites, univariate analysis was carried out, including ANOVA followed by post hoc Tukey test and Benjamini–Hochberg correction at level *α* = 0.05.

Moreover, R Software Package was used to determine Spearman’s correlations at the level of statistical significance *p* < 0.05, between the abundance of microbiota at genus level and gut metabolites.

#### Annotation of metabolites

To identify the compounds after GC–MS analysis, only those metabolites that had a matching mass spectrum of more than 80% with the commercial library NIST Mass Spectral Library were selected and the Kovat retention index (RI) of these compounds, which were calculated using a C10-C40alkane mix (Sigma Aldrich, Germany). Moreover, Table S2 shows the target ion and at least two identifier ions from each metabolite mass spectrum (Table S2).

After UPLC-QTOF-MS analysis, METLIN database provided by the software Agilent Qualitative Analysis Workflow MassHunter B.08.00 was used for the annotation of compounds considering a score higher than 90% and then, LC–MS/MS experiments with the same chromatographic conditions was used to confirm the annotation of some compounds.

In this study, the identified metabolites agreed as level 2 according to MSI (Metabolomics Standards Initiative).

### Microbiota profiling by targeted 16S rRNA gene amplicon–based sequencing

Total DNA was extracted using an automated assisted method based on magnetic beads (Maxwell® RSC Instrument coupled with Maxwell RSC Pure Food GMO and authentication kit, Promega, Spain) following the manufacturer’s instructions and DNA purification (Macherey–Nagel, Duren, Germany) as described previously (Callejón-Leblic et al. 2021). Targeted V3-V4 amplicon library was sequenced on Illumina paired-end platform on a NovaSeq-PE250 Illumina platform (Novogene Bioinformatics Technology Co., Ltd). Controls during DNA extraction and PCR amplification were also included and sequenced.

Raw reads were processed (detailed in Expanded Supplementary Material) using QIIME2 (v. 2018.11) standard 16S workflow (Bolyen et al. 2019). Sequences were denoised with DADA2 (Callahan et al. 2016) with default parameters (tutorial 1.8) and Amplicon Sequence Variants (ASVs) were annotated with RDP database (Martin and Rybicki 2000).

## Results

In our research, an analytical platform based on the complementary use of GC–MS and LC–MS ( ±) has been used to determine the metabolic changes induced in the gut metabolome of *Mus musculus* mice (inbred BALB/c) after DCF exposure and Se supplementation. The combination of both techniques allows to obtain a greater metabolic coverage. This is the first study about the single and joint effects of DCF exposure and Se supplementation on the gut metabolome.

### Survival

**Fig. 1:**
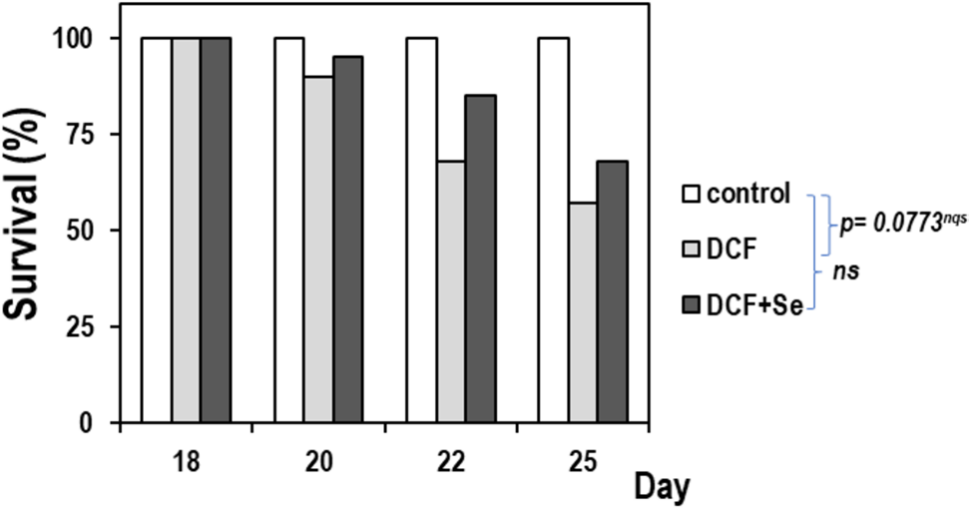
Bar chart showing the percentage of survival after DCF exposure and Se supplementation

A bar chart showing the percentage of survival was carried out to evaluate the survival data in the different exposure groups throughout the experience (Fig. 1). Exposure to DCF triggered the lethality of about 40% of mice at the end of the exposure experience and in Fig. 1 we can see how the percentage of survival decreases as the days progress (15% at 20 days and 30% at 22 days) versus control group. The *t*-Student analysis indicated that mice exposed to DCF had a “not quite significative” lethal data (*p* = 0.0773) versus the control group. In contrast, when mice are exposed to DCF and Se jointly, the lethality percentage was reduced to 30% approximately, not showing significant differences with the control group.

### Speciation of selenoproteins in mice plasma

The selenoproteins quantified in this study were glutathione peroxidase (GPx), selenoproteins P (SELENOP), and selenoalbumin (SeAlb) as well as total selenometabolites (Table 1). A one-way ANOVA followed by the Tukey test was carried out to determine the statistical significance differences between three studied groups. Figure S1 shows the response (increase or decrease) of only those significant comparisons (*p* < 0.05) of the concentration of selenoproteins and selenometabolites in the studied groups. In this figure, a comparison with an additional group (mice supplemented with Se (C-Se)) with the results obtained in our previous study has been included (Callejón-Leblic et al. 2021) (shaded area in Fig. S1).

**Table 1:** Average concentration of selenium (ng of Se per g of plasma) in selenoproteins plasma mice. LOD: detection limit of selenometabolites 0.5 ng Se g^−1^

	**Control**	**DCF**	**DCF-Se**
GPx	12.34 ± 1.46	15.56 ± 1.01	16.36 ± 2.43
Selenometabolites	4.41 ± 0.52	0.62 ± 0.34	1.59 ± 0.21
SELENOP	142.58 ± 16.13	192.41 ± 14.22	208.02 ± 14.39
SeAlb	4.33 ± 0.42	2.86 ± 0.01	4.79 ± 0.87
Total Se	163.66 ± 18.51	211.45 ± 15.24	230.76 ± 17.83

As can be seen in Table 1, the group exposed to DCF and supplemented with Se is the one that shows a higher concentration of total Se. Moreover, in all groups, the distribution of Se follows the order: SELENOP > GPx > Selenometabolites ~ SeAlb. Our previous study showed a significant increase in the concentration of GPx, and SeAlb in the plasma of Se-supplemented mice in comparison with the control group. Exposure to DCF only showed significant changes in the concentration of selenometabolites and Se supplementation partially restored the change in selenometabolites caused by DCF exposure to control levels.

### Untargeted metabolomics

**Fig. 2:**
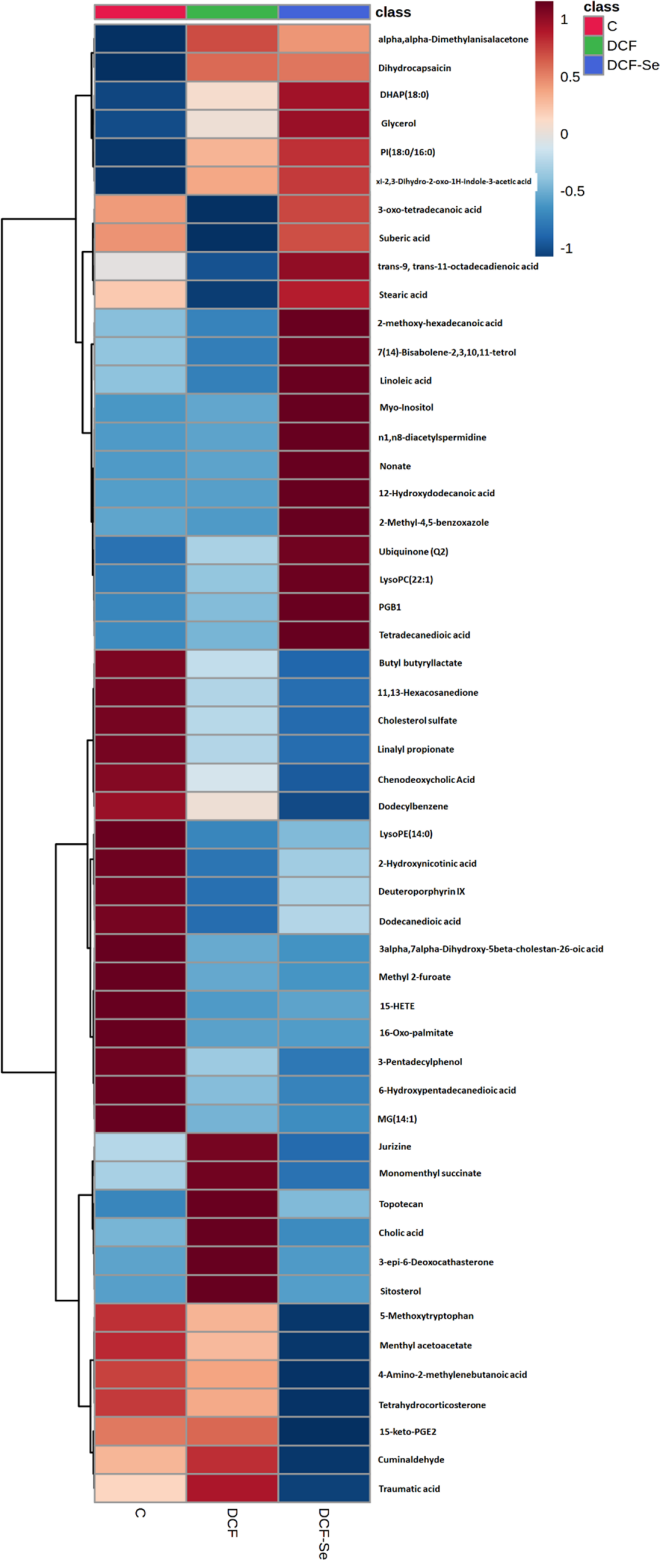
Heatmap showing the mean abundance of each group of significantly identified metabolites after DCF exposure and both, DCF exposure and Se supplementation in mice

A total of 53 gut metabolites were significantly different in abundance by comparing both groups exposed to DCF, fed rodent diet and Se-supplemented diet, vs the control group, combining both analytical techniques, GC–MS (6 metabolites) and UPLC-QTOF ( ±)-MS (48 metabolites). A heatmap summarizing the significant up- and down-regulation of metabolites in gut samples is shown in Fig. 2 and they are listed in Table S3 along their *p*-values and fold changes. These metabolites belong to eighteen different families, where we found 42 altered metabolites in mice exposed to DCF vs control and 38 altered metabolites in mice exposed to DCF and supplemented with Se vs control. The highest number of metabolites belongs to the family of fatty acyls (11 metabolites in DCF group vs control and 10 metabolites in DCF-Se group vs control). In both exposure groups, with and without Se supplementation, the most altered classes of compounds are the fatty acyls, showing decreased levels when compared to the control group, including stearic acid (0.38-fold change in DCF group), dodecanedioic acid (0.40-fold change in DCF group and 0.59-fold change in DCF-Se group), trans-9, trans-11-octadecadienoic acid (0.05- fold change in DCF group), 16-Oxo-palmitate (0.38- fold change in DCF and DCF-Se groups), 3-oxo-tetradecanoic acid (0.62-fold change in DCF group), butyl butyryllactate (0.59-fold change in DCF group and 0.36-fold change in DCF-Se group), and suberic acid (0.78-fold change in DCF group). When the mice are exposed only to DCF, a general tendency can be observed related with an incensement of some metabolites belonging to the family of steroids and derived steroids, such as sitosterol (1.21-fold change), campesterol (1.54-fold change), cholic acid (4.98-fold change), (3beta,5alpha,24S)-Stigmata-7,25-dien-3-ol (2.01-fold change), and 3-epi-6-deoxocathasterone (1.60-fold change). These metabolites show lower abundance (lower fold change) or even restored (non-significant change) when mice are exposed to DCF together with Se.

Previously, the multivariate statistical analysis (PCA) showed a good clustering of quality control samples (QCs), demonstrating a good analytical signal for all analytical platforms used in this study (Fig. S2). On the other hand, 3D-PLS-DA showed good group clustering and classification in GC–MS and UPLC-QTOF ( ±)-MS metabolomic platform (Fig. S3), so *Mus musculus* mice suffer gut metabolic disturbances promoted by DCF exposure and Se supplementation. So, significant differences were found in pairwise comparisons between DCF or DCF-Se vs C (Fig. S4) and this separation between groups indicates that the variables used (m/z of the mass spectra obtained) are capable of discriminating the samples of intestinal content of the mice depending on the treatment received and the next step was to identify the metabolites.

After the analysis, there are gut metabolites that increase considerably their abundance after DCF exposure (Fig. 2), including monomenthyl succinate, topotecan, cholic acid, 3-epi-6-deoxocathasterone, and sitosterol; but their abundance decreases after Se supplementation, being partially restored to the levels of the control group. Otherwise, there is a band of metabolites that decreased in the gut content after DCF exposure that increased after Se supplementation to levels closer to those found in the control group, namely 3-oxo-tetradecanoic acid, suberic acid, trans-9, trans-11-octadecanoic acid, and stearic acid.

The highest number of altered metabolites in the gut metabolome of *Mus musculus* mice was found after DCF exposure and Fig. S5 shows the number of common and different altered metabolites after DCF exposure and DCF plus Se vs control group. Table S4 summarizes the list of metabolites.

These metabolites belonged to sixteen different categories in both groups of exposure, although the proportions of each family are different in the exposure groups (Fig. S6). In both exposure groups, the largest number of metabolites belongs to the family of fatty acyls (26%), followed by steroids and derivatives, being 21% in mice exposed to DCF and 16% in mice exposed to DCF and supplemented with Se. In addition, in both groups we can observe other families such as prenol lipids, glycerophospholipids, glycerolipids, and organooxygen compounds among others.

**Fig. 3:**
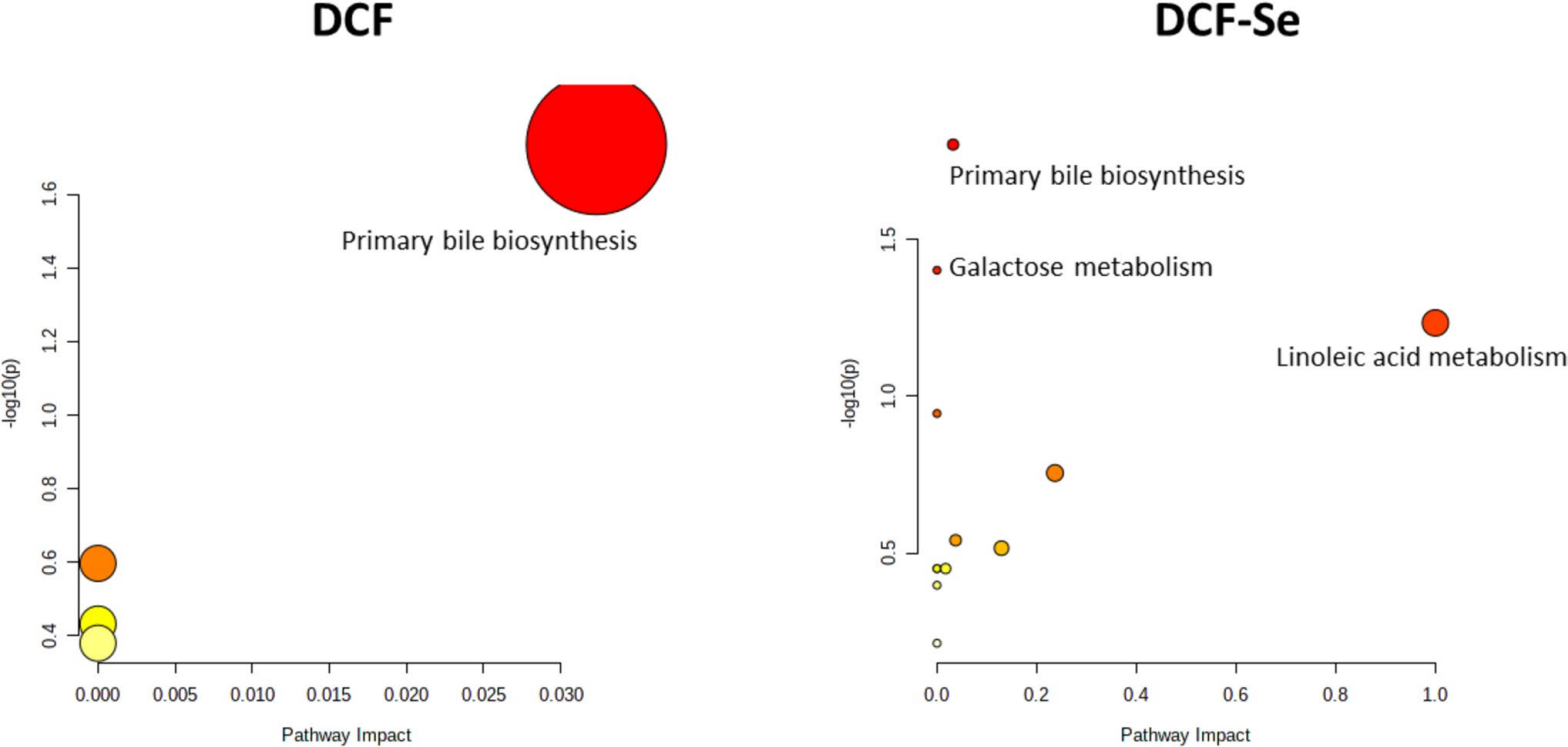
Pathway analysis of altered gut metabolites after DCF and Se exposure. Metabolomic pathway analysis using the KEGG database and generated by MetaboAnalyst 6.0. The color of each circle is based on *p*-values (darker colors indicate more significant changes of metabolites in the corresponding pathway), whereas the size of the circle corresponds to the pathway impact score

Pathway analysis obtained by the metaboanalyst web tool (Fig. 3) was used to identify the altered metabolic pathways after DCF exposure and Se supplementation.

As can be seen, the most significant altered pathway in both groups is the primary bile biosynthesis, being the only pathway altered in mice exposed to DCF, while in mice exposed to DCF and supplemented with Se, the metabolism of galactose and linoleic acid is also altered. Table S5 summarizes *p*-values and pathway impact scores for the most significant pathways.

### Microbiota profiling and associations with specific metabolites

**Fig. 4:**
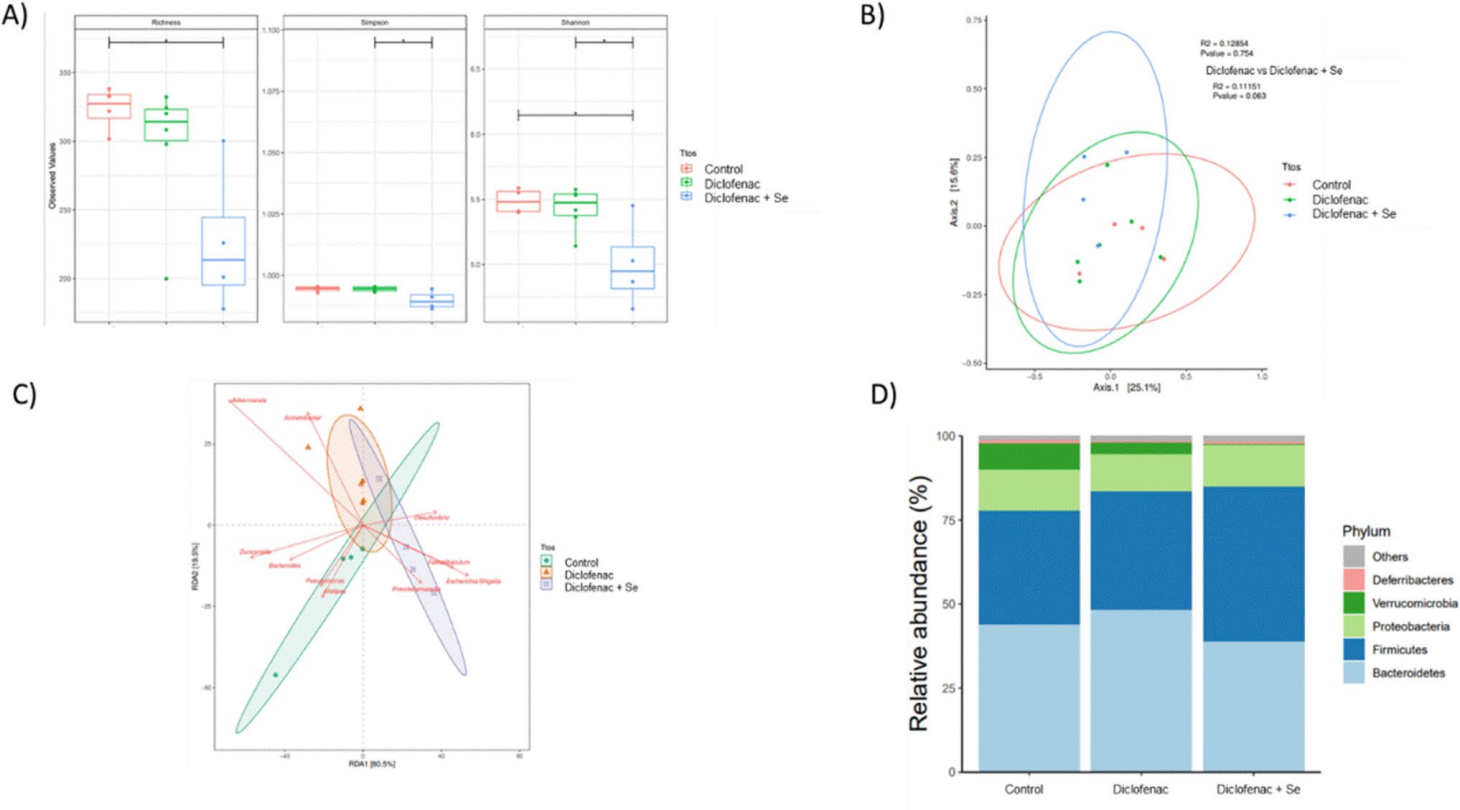
Effect of DCF and Se supplementation on mice microbiota. **A** Alpha diversity of the gut microbial communities according to groups measured as richness (Chao1 and Simpson) and diversity (Shannon) indexes. **B** Principal coordinate analysis (PCoA) of microbial beta-diversity based on the Bray Curtis distance. **C** Redundancy discriminant analysis (RDA) showing the clustering of the mice according to the differences in microbiota composition among groups. **D** Bar plots showing the microbiota composition at phylum level in relative abundance according to groups

Our research shows alterations in the gut microbiota composition and diversity in mice exposed to DCF and mice exposed to DCF fed Se-supplemented diet vs the control group (Fig. 4). Differences in Firmicutes (*p*-value = 0.032, FDR *p*-value > 0.05), Bacteroidetes (*p*-value = 0.015, FDR *p*-value = 0.07), and Verrucomicrobiota (*p*-value = 0.018, FDR *p*-value = 0.08) were observed between groups. Significant differences were also observed in microbial richness and diversity between DCF and DCF-Se fed mice groups (Fig. 4, panel A), being an increase on microbial diversity and richness in DFC diet compared to DFC + Se diet. However, we did not observe significant differences in beta-diversity (PERMANOVA *R*^2^ = 0. 128 and *p*-value = 0.754) using Bray Curtis distance between groups (Fig. 4, panel B), although we observed a trend in the beta-diversity paired comparison between DCF and DFC-Se (PERMANOVA *R*^2^ = 0.111 and *p*-value = 0.063).

A redundancy discriminant analysis (RDA) showed that DFC-Se diet group was associated positively to *E. coli/Shigella*, *Faecalibaculum*, and *Prevotella *genus and negatively with many microbial genus including *Akkermansia* and *Acinetobacter* among others.

**Fig. 5:**
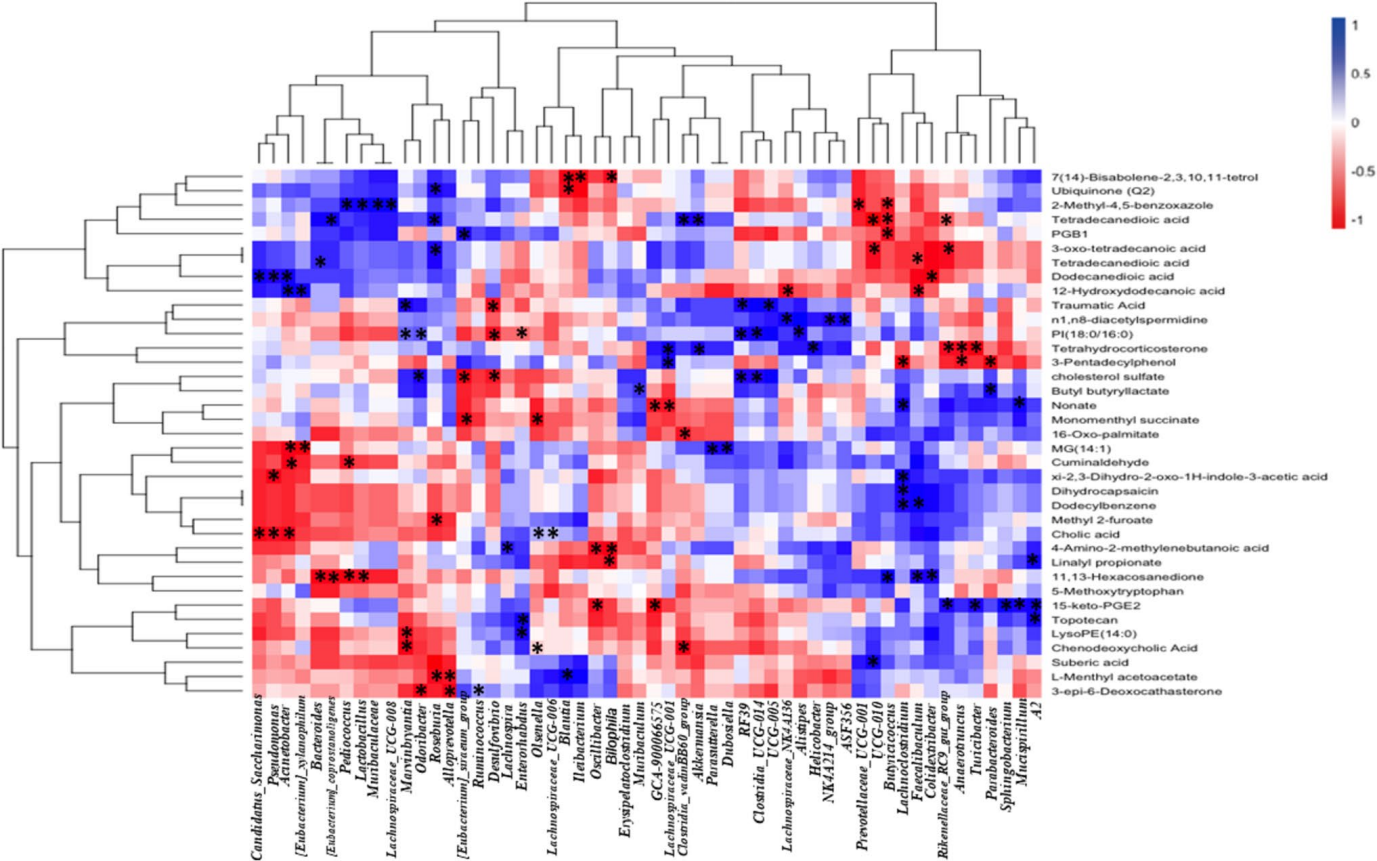
Spearman’s correlation heatmap analysis between altered metabolites and gut microbiota at genus level in *Mus musculus* mice exposed to DCF. The asterisks indicate significant associations. Red color: negative correlation. Blue color: positive correlation. LPC, Lysophosphatidylcholine; LPE, lysophosphatidylethanolamine; PI, phosphatidylinositol; HETE, hydroxyeicosatetraenoic acids; PGB1, prostaglandin B1; MG, monoglyceride

To evaluate the specific associations between gut microbes with gut metabolites, a correlation analysis was carried out. Although we analyzed all of the groups, the analysis of correlations between metabolites and gut microbiota only showed significant associations in mice exposed to DCF (Fig. 5, Table S6).

**Fig. 6:**
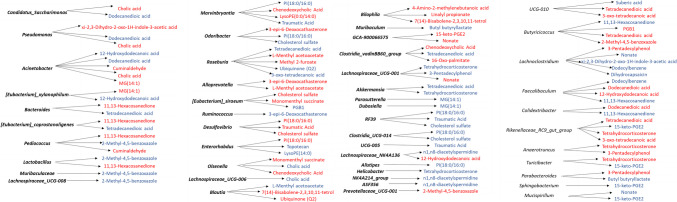
Metabolomics and microbiota results to illustrate the microbiota-metabolome interactions found. Red color: negative correlation, Blue color: positive correlation. LPC, lysophosphatidylcholine; LPE, lysophosphatidylethanolamine; PI, phosphatidylinositol; HETE, hydroxyeicosatetraenoic acids; PGB1, prostaglandin B1; MG, monoglyceride

Moreover, Fig. 6 combines metabolites and microbiota results to illustrate the microbiota-metabolome significant interactions found.

## Discussion

In our previous study, supplementation with Se significantly increased the concentration of the selenoproteins GPx and SeAlb in plasma, without implying a significant change in the concentration of selenometabolites and SELENOP (Callejón-Leblic et al. 2021). These higher levels of the selenoproteins imply a greater availability of Se in *Mus musculus* mice. In the present work, exposure to DCF significantly decreased the concentration of selenometabolites compared to the control group. However, when the mice are exposed to DCF and supplemented with Se, there are no significant differences in the concentrations of selenoproteins versus mice exposed to DCF, although we can observe an increase in selenometabolites levels, which may suggest a potential protective role of Se. Although the level of selenometabolites is closer to that in the control group, when the mice are simultaneously exposed to DCF and Se, a decrease in selenometabolites levels is still observed versus the control group, with no significant differences in the concentration of the selenoproteins.

In both DCF-exposed groups, with and without Se supplementation, the most altered classes of compounds were the fatty acyls, showing decreased levels when compared to the control group. Likewise, the pathways analysis (Fig. 3) showed primary bile biosynthesis as the only altered metabolic pathway in mice exposed to DCF and as one of the major metabolic pathways in mice exposed to DCF and supplemented with Se. Bile acids are products derived from cholesterol in lipid metabolism, which regulate glucose homeostasis, lipid and lipoprotein metabolism, intestinal motility, bacterial growth, inflammation, liver regeneration, and hepatocarcinogenesis (De Aguiar Vallim et al. 2013). Bile acids are of key importance in the metabolism of ingested lipids, interacting with gut bacteria metabolism, through molecular signals, that led to a modification in the abundance and the diversity of the gut microbiota. Other authors have described different impairments in the transformation of primary to secondary bile acids, and how these changes modify the composition of the gut microbiota, which generates processes of chronic inflammation (Collins et al. 2023). Our results show a decrease in the levels of two intermediates involved in the biosynthesis of primary bile acids after DCF exposure, such as 3-alpha,7-alpha-dihydroxy-5-beta-cholestan-26-oic acid (0.38-fold change) and chenodeoxycholic acid (0.50-fold change).

On the other hand, other authors have demonstrated the effect of gut microbiota on long-chain fatty acids (LCFAs) metabolism (Machate et al. 2020). In our study, a decrease of 2-methoxy-hexadecanoic acid (0.60-fold change) was found in mice exposed to DCF vs control (Table S3), while an increase in the level of this metabolite is observed when mice are also supplemented with Se (2.95-fold change). This fact demonstrates the influence of Se supplementation in LCFA metabolism.

A decrease in the levels of deuteroporphyrin IX is found when mice are exposed to DCF and DCF with Se supplementation. Deuteroporphyrin IX is a non-natural dicarboxylic porphyrin described as a fecal porphyrin in patients with endemic chronic arsenic poisoning. Excessive accumulation of the biosynthetic intermediate protoporphyrin can lead to extensive tissue damage upon exposure to light since protoporphyrin is a potent photosensitizing agent, giving rise to membrane-destroying oxygen radicals or singlet oxygen.

### DCF impact of gut metabolome antagonized by Se supplementation

Figure 2 shows some metabolites that are partially restored to control levels after exposure to Se together with DCF. Topotecan increased after DCF exposure (1.11-fold change). Topotecan works by blocking the action of enzyme in cells called topoisomerases, which are necessary for cell replication and tumor growth (El Houari et al. 2022). Blocking this enzyme causes breaks in the DNA, which causes the destruction of cancer cells. In the same way, monomenthyl succinate belonging to the family of prenol lipids increased in DCF group (2.52-fold change) while values were totally restored after exposure to DCF together with Se. Moreover, it can be observed an increase in the abundance of some steroids and steroid derivates such as sitosterol (1.21-fold change), cholic acid (4.98-fold change), and 3-pi-6-deoxocathasterone (1.60-fold change). However, when mice were exposed to Se together with DCF, the abundance of cholic acid decreased (0.48-fold change) versus the control group. In our previous study (Callejón-Leblic et al. 2022), we reported a significant increase of steroids in the gut metabolome of Se-supplemented mice. Cholic acid is a fundamental primary bile acid; it is one of the two major bile acids produced by the liver, where it is synthesized from cholesterol. In the opposite way than cholesterol, cholic acid downregulates cholesterol-7-α-hydroxylase, which is a rate-limiting step in bile acid synthesis. Bile acids perform important physiological detergents that facilitate excretion, absorption, and transport of fats and sterols in the intestine and liver (Collins et al. 2023). Several authors have shown that bile acids have toxic properties when they are present at high levels, could act as a hepatotoxin, damaging the liver or liver cells, or such as a metabotoxin, generating an endogenous metabolite with adverse health effects (Rodrigues et al. 2004).

On the other hand, it can be observed in Fig. 2 a band of metabolites belonging to the family of fatty acyls, as stearic acid, 3-oxo-tetradecanoic acid, and suberic acid, that decreases after exposure to DCF, while the abundance values of these metabolites are closer to the values of the control group when the mice are exposed to Se together with DCF. Fatty acyls are a group of lipids that have important roles as biological signaling, energy storage, cell membrane formation, and protein modification (Samovski et al. 2023). Fatty acids are the most prominent type of fatty acyl. In our previous study (Callejón-Leblic et al. 2022), we reported a significant decrease of fatty acids, their conjugates and monoglycerides in the gut metabolome of Se-supplemented mice.

### DCF impact of gut microbiota affected by Se supplementation

We observed an increase in the abundance of Firmicutes and a decrease in Bacteroidetes phylum in mice exposed to DCF and Se compared to the control group, as previously reported in mice exposed to other NSAIDs such as indomethacin (Xiao et al. 2017). On the other hand, the Proteobacteria phylum increased significantly when mice were simultaneously exposed to DCF and Se compared to the control group. An increased abundance of Proteobacteria in rats after exposure to DCF has been reported by other authors (Colucci et al. 2018), who associate it with DCF-induced enteropathy. Similarly, a recent study has shown that exposure to DCF increases the number of gram-negative bacteria in the ileum of rats and increases intestinal permeability and ulceration (Reuter et al. 1997). In our previous study (Callejón-Leblic et al. 2021), Proteobacteria were higher in the Se-supplemented mice compared to the control, showing an increase in this pro-inflammatory phylum (Rizzatti et al. 2017). In the same study, no differences were found between groups in the main phyla such as Firmicutes, Bacteroides, and Verrucomicrobia. However, other studies have reported significant differences in Firmicutes levels in mice fed Se-supplemented diet (Yu et al. 2020).

### Associations between gut microbes and gut metabolites

In recent years, the study of the microbiota has acquired great importance given its implication in health outcomes. Our research only found associations between gut microbiota and metabolites in mice exposed to DCF fed rodent diet, whereas no associations were found when mice are exposed to DCF and supplemented with Se. *Candidatus-Saccharimonas* were negatively correlated cholic acid (*p*-value = 175, FDR *p*-value = 0.752). Other authors have demonstrated the association of *Candidatus-Saccharimonas* with renal function parameters, and are thus promising biomarkers of renal dysfunction after antibiotic cocktails exposure (Xu et al. 2021). In our study, *Lactobacillus* was positively correlated with the gut metabolite 2-methyl-4,5-benzoxazole (*p*-value = 0.136, FDR *p*-value = 0.569) and negatively correlated with 11,13-hexacosanedione (*p*-value = 175, FDR *p*-value = 0.569), while *Roseburia* was positively correlated with tetradecanedioic acid (*p*-value = 0.497, FDR *p*-value = 0.765), ubiquinone (Q2) (*p*-value = 0.136, FDR *p*-value = 0.765), and 3-oxo-tetradecanoic acid (*p*-value = 0.497, FDR *p*-value = 0.765); and negatively correlated with L-menthyl acetoacetate (*p*-value = 0.241, FDR *p*-value = 0.765) and methyl 2-furoate (*p*-value = 0.297, FDR *p*-value = 0.765). The genus *Lactobacillus* has been linked with effects potentially beneficial on the host, and most of the *Lactobacillus* species have been employed as probiotic (Reid 1999). Interestingly, recent studies carried out by other authors have used *Lactobacillus Rhamnosus UBLR-58* and DCF simultaneously with curcumin to upgrade the effect of the last in the treatment of Alzheimer's disease (Pandel et al. 2021).

In our study, *pseudomonas* show a negative correlation with xi-2,3-dihydro-2-oxo-1H-indole-3-acetic acid (*p*-value = 0.803, FDR *p*-value = 0.967) and cholic acid (*p*-value = 0.419, FDR *p*-value = 0.941) and positive correlation with dodecanedioic acid (*p*-value = 0.803, FDR *p*-value = 0.967). Abbas (2015) demonstrated the inhibitory activity of DCF on the production of virulence factors by *Pseudomonas aeruginosa*.

*Acinetobacter* has a high number of significant correlations with metabolites, being positively associated with 12-hydroxydodecanoic acid (*p*-value = 175, FDR *p*-value = 0.941) and dodecanedioic acid (*p*-value = 0.803, FDR *p*-value = 0.967) and negatively with cuminaldehyde (*p*-value = 0.919, FDR *p*-value = 0.968), cholic acid (*p*-value = 0.419, FDR *p*-value = 0.941), and MG(14:1) (*p*-value = 0.564, FDR *p*-value = 0.941). Previous studies carried out on the crayfish *Procambarus clarki* exposed to DCF have concluded that DCF exposure could affect the intestinal functions including digestion, absorption, and immunity of *Procambarus clarki* by altering the relative abundances of genera including *Acinetobacter* among others, destabilizing the intestinal microbial communities (Zhang et al. 2021). In this regard, these authors also showed an increased abundance of *Bacteroides* after DCF exposure. Bacteroides species are rich in carbohydrate acting enzymes and contain vitamin and glycan (Krinos et al. 2001). Wexler reported that *Bacteroides* could generate distinct capsular polysaccharides to change its surface antigenicity in human colon. Through the interaction with the immune system of host, it can also regulate the atmosphere to affect the growth of some bacteria (Wexler 2007). In our analysis, *Bacteroides* show a positive correlation with tetradecanedioic acid (*p*-value = 0.497, FDR *p*-value = 0.752) and negative correlation with 11,13-hexacosanedione (*p*-value = 0.297, FDR *p*-value = 0.752).

On the other hand, our results show associations in several species of clostridia with metabolites. Clostridia consists of major BSH-producing bacteria, such as *Clostridium* and *Eubacterium*, which are vital in the enterohepatic circulation of bile acids (Song et al. 2019). Thus, *[Eubacterium]_xylanophilum_group* positively correlates with 12-hydroxydodecanoic acid (*p*-value = 0.297, FDR *p*-value = 1) and negatively with MG(14:1) (*p*-value = 0.297, FDR *p*-value = 1); *[Eubacterium]_coprostanoligenes_group* positively correlates tetradecanedioic acid (*p*-value = 0.419, FDR *p*-value = 0.799) and negatively with 11,13-hexacosanedione (*p*-value = 0.242, FDR *p*-value = 0.743); *[Eubacterium]_siraeum_group* positively correlated with PGB1 (*p*-value = 0.919, FDR *p*-value = 0.943) and negatively with cholesterol sulfate (*p*-value = 0.564, FDR *p*-value = 0.892); *Clostridia_vadinBB60_group* positively correlates with tetradecanedioic acid (*p*-value = 1, FDR *p*-value = 1) and negatively with chenodeoxycholic acid (*p*-value = 0.564, FDR *p*-value = 1) and 16-Oxo-palmitate (*p*-value = 0.564, FDR *p*-value = 0.892) and finally *Clostridia_UCG-014* positively correlates with PI(18:0/16:0) (*p*-value = 0.242, FDR *p*-value = 0.865).

Specific correlations between gut metabolites and members from the Lachnospiraceae, Prevotellaceae, and Ruminococcaceae families were identified. Likewise, *Lachnospiraceae_UCG-008* was positively correlated with 2-methyl-4,5-benzoxazole (*p*-value = 0.919, FDR *p*-value = 0.968); *Lachnospiraceae_UCG-006* positively correlated with cholic acid (*p*-value = 0.497, FDR *p*-value = 0.806); *Lachnospiraceae_UCG-001* was positively correlated with tetrahydrocorticosterone (*p*-value = 0.714, FDR *p*-value = 1) and 3-Pentadecylphenol (*p*-value = 0.103, FDR *p*-value = 1) and negatively correlated with nonate (*p*-value = 0.803, FDR *p*-value = 1) and finally *Lachnospiraceae_NK4A136_group* positively correlated with n1,n8-diacetylspermidine (*p*-value = 0.919, FDR *p*-value = 1) and negatively correlated with 12-hydroxydodecanoic acid (*p*-value = 0.297, FDR *p*-value = 1). Moreover, *Prevotellaceae_UCG-001* was negatively correlated with 2-methyl-4,5-benzoxazole (*p*-value = 0.564, FDR *p*-value = 0.805). *Rikenellaceae_RC9_gut_group* shows negative correlations with tetradecanedioic acid (*p*-value = 0.103, FDR *p*-value = 0.411), tetrahydrocorticosterone (*p*-value = 0.103, FDR *p*-value = 0.411), and 3-oxo-tetradecanoic acid (*p*-value = 0.103, FDR *p*-value = 0.411), and positive correlation with 15-keto-PGE2 (*p*-value = 0.658, FDR *p*-value = 0.798).

Finally, *Ruminococcus* was only positively correlated with a steroid compound (3-epi-6-Deoxocathasterone) (*p*-value = 0.564, FDR *p*-value = 1). This genus and other members of the Ruminococcaceae are very important for the protection of gut barrier and maintain intestinal immune homeostasis, by producing SCFAs (Darnaud et al. 2018).

## Conclusions

Our results highlight the impact of DCF on gut metabolome and micorbiota. Se supplementation changed the metabolic and microbiota profiles of the gut content restoring almost partially the composition and abundance of some metabolites. Although Se supplementation modulated gut microbiota increasing some heath-relevant taxa and could antagonize the impact of several pollutants in the gut microbiota, the joined Se and DCF effects led to a decreased diversity indicating a synergistic effect with DCF and limited capacity to counteract the deleterious effects at microbiota level. The most significant altered pathway in DCF and Se-DCF groups is the primary bile biosynthesis, being the only pathway altered in mice exposed to DCF, while in DCF-Se group, the metabolism of galactose and linoleic acid is also altered. Interestingly, we found numerous associations between gut microbes and gut metabolites in all the groups, except in the mice exposed to DCF and fed Se supplementation. Thus, there is a potential key interaction between Se intake–gut microbiota–gut metabolites with potential effects on the host health. However, the precise link needs to be ascertained and further studies targeted to the specific mechanisms are claimed.

## Supplementary Information

Below is the link to the electronic supplementary material.

Supplementary file1 (DOCX 1650 KB)

## Data Availability

Data will be available upon request.

## Abbreviations

C: Control
DCF: Diclofenac
Se: Selenium
GC-MS: Gas chromatography coupled to mass spectrometry
UHPLC-QTOF-MS: Ultra-high performance liquid chromatography-quadrupole time-of-flight mass spectrometry
PACs: Pharmaceutically active compounds
NSAID: Non-steroidal anti-inflammatory drug
MSTFA: *N*-Methyl-*N*-(trimethylsilyl)trifluoroacetamide
MG: Monoradylglycerol
LysoPE: Lysophosphatidylethanolamine
LysoPC: Lysophosphatidylcholine
PGB1: Prostaglandin B1
HETE: Hydroxyeicosatetraenoic acids
LCFA: Long-chain fatty acids

## References

[CR1] Abbas HA (2015) Inhibition of virulence factors of pseudomonas aeruginosa by diclofenac sodium. Roum Arch Microbiol Immunol 74:79–85 27328521

[CR2] Ahlawat S, Shankar A, Mohan H, Sharma KK (2021) Yersinia enterocolitica and Lactobacillus fermentum induces differential cellular and behavioral responses during diclofenac biotransformation in rat gut. Toxicol Appl Pharmacol 431:115741. 10.1016/j.taap.2021.115741 34619158 10.1016/j.taap.2021.115741

[CR3] Aygün D, Kaplan S, Odaci E, Onger M, Altunkaynak M (2012) Toxicity of non-steroidal anti-inflammatory drugs: a review of melatonin and diclofenac sodium association. Histol Histopathol 27(4):417–436 22374720 10.14670/HH-27.417

[CR4] Bolyen E, Rideout JR, Dillon MR, Bokulich NA, Abnet CC, Al-Ghalith GA, Alexander H, Alm EJ, Arumugam M, Asnicar F, Bai Y, Bisanz JE, Bittinger K, Brejnrod A, Brislawn CJ, Brown CT, Callahan BJ, Caraballo-Rodríguez AM, Chase J, Cope EK, Da Silva R, Diener C, Dorrestein PC, Douglas GM, Durall DM, Duvallet C, Edwardson CF, Ernst M, Estaki M, Fouquier J, Gauglitz JM, Gibbons SM, Gibson DL, Gonzalez A, Gorlick K, Guo J, Hillmann B, Holmes S, Holste H, Huttenhower C, Huttley GA, Janssen S, Jarmusch AK, Jiang L, Kaehler BD, Kang KB, Keefe CR, Keim P, Kelley ST, Knights D, Koester I, Kosciolek T, Kreps J, Langille MGI, Lee J, Ley R, Liu YX, Loftfield E, Lozupone C, Maher M, Marotz C, Martin BD, McDonald D, McIver LJ, Melnik AV, Metcalf JL, Morgan SC, Morton JT, Naimey AT, Navas-Molina JA, Nothias LF, Orchanian SB, Pearson T, Peoples SL, Petras D, Preuss ML, Pruesse E, Rasmussen LB, Rivers A, Robeson MS, Rosenthal P, Segata N, Shaffer M, Shiffer A, Sinha R, Song SJ, Spear JR, Swafford AD, Thompson LR, Torres PJ, Trinh P, Tripathi A, Turnbaugh PJ, Ul-Hasan S, van der Hooft JJJ, Vargas F, Vázquez-Baeza Y, Vogtmann E, von Hippel M, Walters W, Wan Y, Wang M, Warren J, Weber KC, Williamson CHD, Willis AD, Xu ZZ, Zaneveld JR, Zhang Y, Zhu Q, Knight R, Caporaso JG (2019) Reproducible, interactive, scalable and extensible microbiome data science using QIIME 2. Nat Biotechnol 37:852–857. 10.1038/s41587-019-0209-9 31341288 10.1038/s41587-019-0209-9 PMC7015180

[CR5] Callahan BJ, McMurdie PJ, Rosen MJ, Han AW, Johnson AJA, Holmes SP (2016) DADA2: high-resolution sample inference from Illumina amplicon data. Nat Methods 13:581–583. 10.1038/nmeth.3869 27214047 10.1038/nmeth.3869 PMC4927377

[CR6] Callejón-Leblic B, Selma-Royo M, Collado MC, Abril N, García-Barrera T (2021) Impact of antibiotic-induced depletion of gut microbiota and selenium supplementation on plasma selenoproteome and metal homeostasis in a mice model. J Agric Food Chem 69:7652–7662. 10.1021/acs.jafc.1c02622 34171188 10.1021/acs.jafc.1c02622 PMC9161447

[CR7] Callejón-Leblic B, Selma-Royo M, Collado MC, Gómez-Ariza JL, Abril N, García-Barrera T (2022) Untargeted gut metabolomics to delve the interplay between selenium supplementation and gut microbiota. J Proteome Res 21:758–767. 10.1021/acs.jproteome.1c00411 34734730 10.1021/acs.jproteome.1c00411 PMC8902802

[CR8] Collins SL, Stine JG, Bisanz JE, Okafor CD, Patterson AD (2023) Bile acids and the gut microbiota: metabolic interactions and impacts on disease. Nat Rev Microbiol 21:236–247. 10.1038/s41579-022-00805-x 36253479 10.1038/s41579-022-00805-x PMC12536349

[CR9] Colucci R, Pellegrini C, Fornai M et al (2018) Pathophysiology of NSAID-associated intestinal lesions in the rat: luminal bacteria and mucosal inflammation as targets for prevention. Front Pharmacol 9:1–14. 10.3389/fphar.2018.01340 10.3389/fphar.2018.01340 PMC6281992 30555323

[CR10] D’Amato A, Di Cesare Mannelli L, Lucarini E, Man AL, Le Gall G, Branca JJ, Ghelardini C, Amedei A, Bertelli E, Regoli M, Pacini A (2020) Faecal microbiota transplant from aged donor mice affects spatial learning and memory via modulating hippocampal synaptic plasticity- and neurotransmission- related proteins in young recipients. Microbiome 8:1–19 33004079 10.1186/s40168-020-00914-w PMC7532115

[CR11] Darnaud M, Dos Santos A, Gonzalez P, Augui S, Lacoste C, Desterke C, De Hertogh G, Valentino E, Braun E, Zheng J, Boisgard R, Neut C, Dubuquoy L, Chiappini F, Samuel D, Lepage P, Guerrieri F, Doré J, Bréchot C, Moniaux N, Faivre J (2018) Enteric delivery of regenerating family member 3 alpha alters the intestinal microbiota and controls inflammation in mice with colitis. Gastroenterology 154:1009-1023.e14. 10.1053/j.gastro.2017.11.003 29133078 10.1053/j.gastro.2017.11.003

[CR12] De Aguiar Vallim TQ, Tarling EJ, Edwards PA (2013) Pleiotropic roles of bile acids in metabolism. Cell Metab 17:657–669. 10.1016/j.cmet.2013.03.013 23602448 10.1016/j.cmet.2013.03.013 PMC3654004

[CR13] El Houari A, Ecale F, Mercier A, Crapart S, Laparre J, Soulard B, Ramnath M, Berjeaud JM, Rodier MH, Crépin A (2022) Development of an in vitro model of human gut microbiota for screening the reciprocal interactions with antibiotics, drugs, and xenobiotics. Front Microbiol 13:1–22. 10.3389/fmicb.2022.828359 10.3389/fmicb.2022.828359 PMC9042397 35495704

[CR14] García-Barrera T, Gómez-Ariza JL, González-Fernández M, Moreno F, García-Sevillano MA, Gómez-Jacinto V (2012) Biological responses related to agonistic, antagonistic and synergistic interactions of chemical species. Anal Bioanal Chem 403:2237–2253. 10.1007/s00216-012-5776-2 22367285 10.1007/s00216-012-5776-2

[CR15] Gomaa S (2018) Adverse effects induced by diclofenac, ibuprofen, and paracetamol toxicity on immunological and biochemical parameters in Swiss albino mice. J Basic Appl Zool 79:1–9. 10.1186/s41936-018-0025-7

[CR16] He Z, Wei G, Li N, Niu M, Gong S, Wu G, Wang T, Jiang Y, Chen P (2019) CCR2 and CCR5 promote diclofenac-induced hepatotoxicity in mice. Naunyn Schmiedebergs Arch Pharmacol 392:287–297. 10.1007/s00210-018-1576-3 30483860 10.1007/s00210-018-1576-3

[CR17] Katajamaa M, Oresic M (2007) Data processing for mass spectrometry-based metabolomics. J Chromatogr A 1158:318–328. 10.1016/j.chroma.2007.04.021 17466315 10.1016/j.chroma.2007.04.021

[CR18] Klampfl CW (2019) Metabolization of pharmaceuticals by plants after uptake from water and soil: a review. TrAC - Trends Anal Chem 111:13–26. 10.1016/j.trac.2018.11.042

[CR19] Krinos CM, Coyne MJ, Weinacht KG, Tzianabos AO, Kasper DL, Comstock LE (2001) Extensive surface diversity of a commensal microorganism by multiple DNA inversions. Nature 414:555–558. 10.1038/35107092 11734857 10.1038/35107092

[CR20] Lonappan L, Brar SK, Das RK, Verma M, Surampalli RY (2016) Diclofenac and its transformation products: environmental occurrence and toxicity - a review. Environ Int 96:127–138. 10.1016/j.envint.2016.09.014 27649472 10.1016/j.envint.2016.09.014

[CR21] Machate DJ, Figueiredo PS, Marcelino G, Guimarães RD, Hiane PA, Bogo D, Pinheiro VA, Oliveira LC, Pott A (2020) Fatty acid diets: regulation of gut microbiota composition and obesity and its related metabolic dysbiosis. Int J Mol Sci 21(1):22. 10.3390/ijms21114093 10.3390/ijms21114093 PMC7312778 32521778

[CR22] Martin D, Rybicki E (2000) RDP: detection of recombination amongst aligned sequences. Bioinformatics 16:562–563 10980155 10.1093/bioinformatics/16.6.562

[CR23] Mayorek N, Naftali-Shani N, Grunewald M (2010) Diclofenac inhibits tumor growth in a murine model of pancreatic cancer by modulation of VEGF levels and arginase activity. PLoS ONE 5:1–10. 10.1371/journal.pone.0012715 10.1371/journal.pone.0012715 PMC2939880 20856806

[CR24] Morais LH, Schreiber HL, Mazmanian SK (2021) The gut microbiota–brain axis in behaviour and brain disorders. Nat Rev Microbiol 19:241–255. 10.1038/s41579-020-00460-0 33093662 10.1038/s41579-020-00460-0

[CR25] Oaks JL, Gilbert M, Virani MZ, Watson RT, Meteyer CU, Rideout BA, Shivaprasad HL, Ahmed S, Chaudhry MJI, Arshad M, Mahmood S, Ali A, Khan AA (2004) Diclofenac residues as the cause of vulture population decline in Pakistan. Nature 427:630–633. 10.1038/nature02317 14745453 10.1038/nature02317

[CR26] Owumi SE, Aliyu-Banjo NO, Odunola OA (2020) Selenium attenuates diclofenac-induced testicular and epididymal toxicity in rats. Andrologia 52:1–11. 10.1111/and.13669 10.1111/and.13669 32510627

[CR27] Pandel S, Patel C, Sarkar D, Acharya S (2021) Lactobacillus Rhamnosus UBLR-58 and diclofenac potentiate the anti-Alzheimer activity of curcumin in mice. Curr Enzym Inhib 17:49–56

[CR28] Peters A, Crane M, Merrington G, Ryan J (2022) Environmental quality standards for diclofenac derived under the European water framework directive: 2 Avian secondary poisoning. Environmental Sciences Europe 34(1):28. 10.1186/s12302-022-00601-7

[CR29] Rastogi A, Tiwari MK, Ghangrekar MM (2021) A review on environmental occurrence, toxicity and microbial degradation of Non-Steroidal Anti-Inflammatory Drugs (NSAIDs). J Environ Manage 300:113694. 10.1016/j.jenvman.2021.113694 34537557 10.1016/j.jenvman.2021.113694

[CR30] Reid G (1999) The scientific basis for probiotic strains of Lactobacillus. Appl Environ Microbiol 65:3763–3766. 10.1128/aem.65.9.3763-3766.1999 10473372 10.1128/aem.65.9.3763-3766.1999 PMC99697

[CR31] Reuter BK, Davies NM, Wallace JL (1997) Nonsteroidal anti-inflammatory drug enteropathy in rats: role of permeability, bacteria, and enterohepatic circulation. Gastroenterology 112:109–117. 10.1016/S0016-5085(97)70225-7 10.1016/s0016-5085(97)70225-7 8978349

[CR32] Rey-Stolle F, Dudzik D, Gonzalez-Riano C, Fernández-García M, Alonso-Herranz V, Rojo D, Barbas C, García A (2022) Low and high resolution gas chromatography-mass spectrometry for untargeted metabolomics: a tutorial. Anal Chim Acta 1210:339043. 10.1016/j.aca.2021.339043 35595356 10.1016/j.aca.2021.339043

[CR33] Rizzatti G, Lopetuso LR, Gibiino G et al (2017) Proteobacteria: a common factor in human diseases. Biomed Res Int. 10.1155/2017/935150 10.1155/2017/9351507 PMC5688358 29230419

[CR34] Rodrigues CMP, Castro RE, Steer CJ (2004) 5. The role of bile acids in the modulation of apoptosis. Princ Med Biol 15:119–145. 10.1016/S1569-2582(04)15005-8

[CR35] Samovski D, Jacome-Sosa M, Abumrad NA (2023) Fatty acid transport and signaling: mechanisms and physiological implications. Annu Rev Physiol 85:317–337. 10.1146/annurev-physiol-032122-030352 36347219 10.1146/annurev-physiol-032122-030352 PMC13221695

[CR36] Sathishkumar P, Mohan K, Meena RAA, Balasubramanian M, Chitra L, Ganesan AR, Palvannan T, Brar SK, Gu FL (2021) Hazardous impact of diclofenac on mammalian system: mitigation strategy through green remediation approach. J Hazard Mater 419:126135. 10.1016/j.jhazmat.2021.126135 34157463 10.1016/j.jhazmat.2021.126135

[CR37] Selvaraj S, Oh JH, Yoon S, Borlak J (2023) Diclofenac disrupts the circadian clock and through complex cross-talks aggravates immune-mediated liver injury—a repeated dose study in minipigs for 28 days. Int J Mol Sci 24:1445. 10.3390/ijms24021445 36674967 10.3390/ijms24021445 PMC9863319

[CR38] Song Z, Cai Y, Lao X, Wang X, Lin X, Cui Y, Kalavagunta PK, Liao J, Jin L, Shang J, Li J (2019) Taxonomic profiling and populational patterns of bacterial bile salt hydrolase (BSH) genes based on worldwide human gut microbiome. Microbiome 7:1–16. 10.1186/s40168-019-0628-3 30674356 10.1186/s40168-019-0628-3 PMC6345003

[CR39] Thai PN, Ren L, Xu W, Overton J, Timofeyev V, Nader CE, Haddad M, Yang J, Gomes AV, Hammock BD, Chiamvimonvat N, Sirish P (2023) Chronic diclofenac exposure increases mitochondrial oxidative stress, inflammatory mediators, and cardiac dysfunction. Cardiovasc Drugs Ther 37:25–37. 10.1007/s10557-021-07253-4 34499283 10.1007/s10557-021-07253-4 PMC8904649

[CR40] Wexler HM (2007) Bacteroides: the good, the bad, and the nitty-gritty. Clin Microbiol Rev 20:593–621. 10.1128/CMR.00008-07 17934076 10.1128/CMR.00008-07 PMC2176045

[CR41] Xiao X, Nakatsu G, Jin Y et al (2017) Gut microbiota mediates protection against enteropathy induced by indomethacin. Sci Rep 7:1–11. 10.1038/srep40317 10.1038/srep40317 PMC5220306 28067296

[CR42] Xu J, Xu HM, Peng Y, Zhao C, Zhao HL, Huang W, Huang HL, He J, Du YL, Zhou YJ, Zhou YL (2021) The effect of different combinations of antibiotic cocktails on mice and selection of animal models for further microbiota research. Appl Microbiol Biotechnol 105(1669):1681. 10.1007/s00253-021-11131-2 10.1007/s00253-021-11131-2 33511441

[CR43] Yu T, Guo J, Zhu S et al (2020) Protective effects of selenium-enriched peptides from: cardamine violifolia against high-fat diet induced obesity and its associated metabolic disorders in mice. RSC Adv 10:31411–31424. 10.1039/d0ra04209a 10.1039/d0ra04209a PMC9056391 35520651

[CR44] Zádori ZS, Király K, Al-Khrasani M, Gyires K (2023) Interactions between NSAIDs, opioids and the gut microbiota - future perspectives in the management of inflammation and pain. Pharmacol Ther 241:108327. 10.1016/j.pharmthera.2022.108327 36473615 10.1016/j.pharmthera.2022.108327

[CR45] Zarrinpar A, Chaix A, Xu ZZ, Chang MW, Marotz CA, Saghatelian A, Knight R, Panda S (2018) Antibiotic-induced microbiome depletion alters metabolic homeostasis by affecting gut signaling and colonic metabolism. Nat Commun 9(1):2872. 10.1038/s41467-018-05336-9 30030441 10.1038/s41467-018-05336-9 PMC6054678

[CR46] Zhang Y, Sun K, Li Z, Chai X, Fu X, Kholodkevich S, Kuznetsova T, Chen C, Ren N (2021) Effescts of acute diclofenac exposure on intestinal histology, antioxidant defense, and microbiota in freshwater crayfish (Procambarus clarkii). Chemosphere 263:128130. 10.1016/j.chemosphere.2020.128130 33297118 10.1016/j.chemosphere.2020.128130

