# Supplementary material for: Modulation of the gut microbiota and the microbial-produced gut metabolites by diclofenac exposure and selenium supplementation
Source: Environ Sci Pollut Res Int. 2025 Mar 18;32(28):16945–57. doi: 10.1007/s11356-025-36233-6 (PMC12325468; doi:10.1007/s11356-025-36233-6)
Supplement: Supplementary file 1 — Supplementary file1 (DOCX 1650 KB) [file 11356_2025_36233_MOESM1_ESM.docx]

**SUPPORTING INFORMATION**

**Modulation of the Gut Microbiota and the Microbial-Produced Gut Metabolites by Diclofenac Exposure and Selenium Supplementation**

Gema Rodríguez-Moro^a^, Raúl Cabrera-Rubio^b^, Marta Selma-Royo^b^, José Antonio Gómez-Morlote^a^, Maria Carmen Collado^bΣ^, Nieves Abril^cΣ^, Tamara García-Barrera^aΣ*^

*^a^Research Center of Natural Resources, Health and the Environment (RENSMA). Department of Chemistry, Faculty of Experimental Sciences, University of Huelva, Fuerzas Armadas Ave., 21007, Huelva, Spain; ^b^Institute of Agrochemistry and Food Technology (IATA-CSIC), Department of Biotechnology, Agustin Escardino 7. 46980 Paterna, Valencia, Spain, ^c^Department of Biochemistry and Molecular Biology, University of Córdoba, Campus de Rabanales, Edificio Severo Ochoa, E-14071, Córdoba, Spain. Σ senior authors; *tamara@dqcm.uhu.es*

**2. Experimental section**

**2.2.2.** **SEC-AF-SUID-ICP-QQQ-MS experimental conditions**

100 μL of plasma was injected into a high-performance liquid chromatograph model 1260 Infinity Quaternary LC (Agilent Technologies) connected to two 5 mL HiTrap Desalting Columns (GE Healthcare, Uppsala, Sweden) and two affinity columns of heparin-sepharose (HEP-HP) and blue-sepharose (BLU-HP) (GE Healthcare, Uppsala, Sweden). Ammonium acetate was used for the preparation of mobile phases A (0.05 M, pH = 7.4) and B (1.5 M, pH = 7.4) and the flow-rate was set at 1.3 mL min^−1^. The columns were interconnected using a six-way valve and finally, they were coupled to a triple quadrupole inductively coupled plasma mass spectrometer model Agilent 8800 Triple Quad (Agilent Technologies, Tokyo, Japan) through a Micromist nebulizer (Glass Expansion, Switzerland). The HEP-HP column is able to retain selenoprotein P (SEPP1), while the BLU-HP column retains both SEPP1 and selenoalbumin (SeAlb). To separate the selenoproteins, we applied two working modes: (i) Mode 1 (from 0 to 20 min, mobile phase A); the plasma simple passes through the whole system 2D-SEC-SEC-AF(HEP-HP)xAF- (BLU-HP)-ICP-MS, allowing the elution of plasma glutathione peroxidase (GPx) and selenometabolites at 4 and 8 min, respectively, and the retention of SEPP1 in the HEP-HP column and SeAlb in BLU-HP column; (ii) Mode 2 (from 20 to 24 min, mobile phase B); SEPP1 elutes at 20.5 min and SeAlb is isolated in the BLU-HP column; (iii) Mode 3 (from 24 to 40 min, mobile phase B); SeAlb is released and can elute at 25 min. The absolute quantification of selenocompounds was carried out using the species unspecific isotopic dilution analysis. To this end, a flow-rate of 0.1 mL min−1 of Seenriched standard (^74^Se Cambridge Isotope Laboratories, Andover, MA, USA) was introduced into the system after the chromatographic separation (post-column) using a T shape connector. The quality of the analytical method was verified using the human serum BCR-637 CRM (Institute for Reference Materials and Measurements, IRMM, Geel, Belgium).

**2.3.2. GC-MS experimental conditions**

Metabolomic analysis by GC-MS was performed using a Factor Four VF-5MS column (30 m × 0.25 μm ID, 0.25 μm of film thickness) on a Trace GC ULTRA gas chromatograph coupled to an ion trap mass spectrometer detector ITQ900 (Thermo Fisher Scientific). A volume of 1 µL of the extract was injected into the column in splitless mode. The analyses were carried out in full scan mode with the mass range of 35-650 m/z and electron impact ionization (EI) with a voltage of 70 eV. The injector temperature was maintained at 280 ºC using helium as carrier gas at 1 mL·min^−1^. The chromatographic method was 50 ºC for 1 min, then programmed to increase to 310 ºC at 10 ºC·min^−1^, and finally maintained for 10 min. The filament was off in the first 6 min of the chromatogram to avoid the signal of the solvent. Tricosan was used as internal standard.

**2.3.3. UPLC-QTOF-MS conditions**

For UPLC-QTOF-MS experiments, the extracts were analyzed using an Agilent 6550 iFunnel Q-TOF LC/MS system (Agilent Technologies, Tokyo, Japan) equipped with a dual electrospray ion source for positive and negative ionization modes, coupled to two binary pumps and thermostatic autosampler Agilent 1290 Series LC pump with inverse phase chromatography. An aliquot of 100 μL of the extract was taken and placed in an autosampler at 4 ºC, and 10 μL was taken for injection into an EclipsePlusC18 RRHD column (50 mm × 2.1 mm, 1.8 μm) thermostasted at 40 ºC. The mobile phase flow was set at 0.4 mL·min^−1^ with solvent A (0.1% (v/v) formic acid in water) and solvent B (0.1% (v/v) formic acid in acetonitrile). The chromatographic separation gradient started at 5% solvent B for 1 min, increasing to 95% in 13 min, which was maintained for 3 min, getting back to the starting conditions in 26 min and held for 4 min. The total time of analysis was 30 minutes. During the analysis, two reference masses were parallel injected into the system for mass correction: 121.0509 and 922.0098 m/z for positive ionization and m/z 112.9856 and 1033.9881 m/z for negative ionization. Optimized system parameters were: full scan mode from 100 to 1100 m/z, drying gas flow rate 12 L min^-1^ at 250 ºC, gas nebulizer at 35 psi, fragmentor voltage at 175 V for positive mode and 250 V for negative mode, capillary voltage set to 380 V and 750 V octopolar radiofrequency. Data were acquired in centroid mode at scan ratio of 1 spectra·s^-1^.

**2.4. Microbiota profiling by targeted 16S rRNA amplicon-based sequencing**

**DNA extraction and *16S rRNA amplicon sequencing***

Total DNA was extracted from paired intestinal contents material (approx. 100 mg) using an automated assisted method based on magnetic beads (Maxwell® RSC Instrument coupled with Maxwell RSC Pure Food GMO and authentication kit, Promega, Spain) following the manufacturer’s instructions with previous treatments to improve the DNA quality and efficiency. In brief, samples were treated with lysozyme (20 mg/mL) and mutanolysin (5 U/mL) for 60 min at 37 °C and a preliminary step of cell disruption with 3-μm diameter glass beads during 1 min at 6 m/s by a bead beater FastPrep 24-5G Homogenizer (MP Biomedicals). DNA obtained was purified by use of DNA Purification Kit (Macherey-Nagel, Duren, Germany) according to manufacturer’s instructions and DNA concentration was measured using Qubit® 2.0 Fluorometer (Life Technology, Carlsbad, CA, US) for further analysis.

Microbial profiles were determined by V3-V4 variable region of the 16S rRNA gene sequencing following PE250 Illumina strategy sequencing protocols. Amplicons were obtained with PCR-amplified using barcoded conventional primers (341F 5′-CCTACGGGNGGCWGCAG-3′ and 806R 5′GGACTACNNGGGTATCTAAT-3′). The libraries were generated with NEBNext® UltraTM DNA Library Prep Kit for Illumina and amplicons were checked with a Bioanalyzer DNA 1000 chip (Agilent Technologies, Santa Clara, CA, United States). Amplicon was sequenced on Illumina paired-end platform to generate 250 bp paired-end raw reads on a NovaSeq- PE250 Illumina platform (Novogene Bioinformatics Technology Co., Ltd) according to manufacturer instructions. Controls during DNA extraction and PCR amplification were also included and sequenced.

**Microbiota sequences analysis**

Bacterial diversity analysis was done using raw reads, which were quality controlled and filtered (Q > 20 and length > 100 bp) using fastqc (v0.11.8) and trimGalore (v0.6.4_dev; <https://github.com/FelixKrueger/TrimGalore>). In addition, trimGalore was used for adapter removal. The reads resulting from the previous process were processed using the Quantitative Insights Into Microbial Ecology 2’s (QIIME2) (version 2018.11) standard 16S workflow was used for analysis. Sequences were denoised with DADA2 run with default parameters (tutorial 1.8). Amplicon Sequence Variants (ASVs) were annotated with a Naïve-Bayes classifier based on the scikit-learn system and the RDP database. The ASVs were aligned with MAFFT, to then make a phylogenetic tree with FASTTREE that was then midpoint-rooted.

**3. Results**

**Figure S1.** Comparative diagram of the response of the selenoproteins concentration between the studied groups. N.S: no significant changes. * data from our previous study.


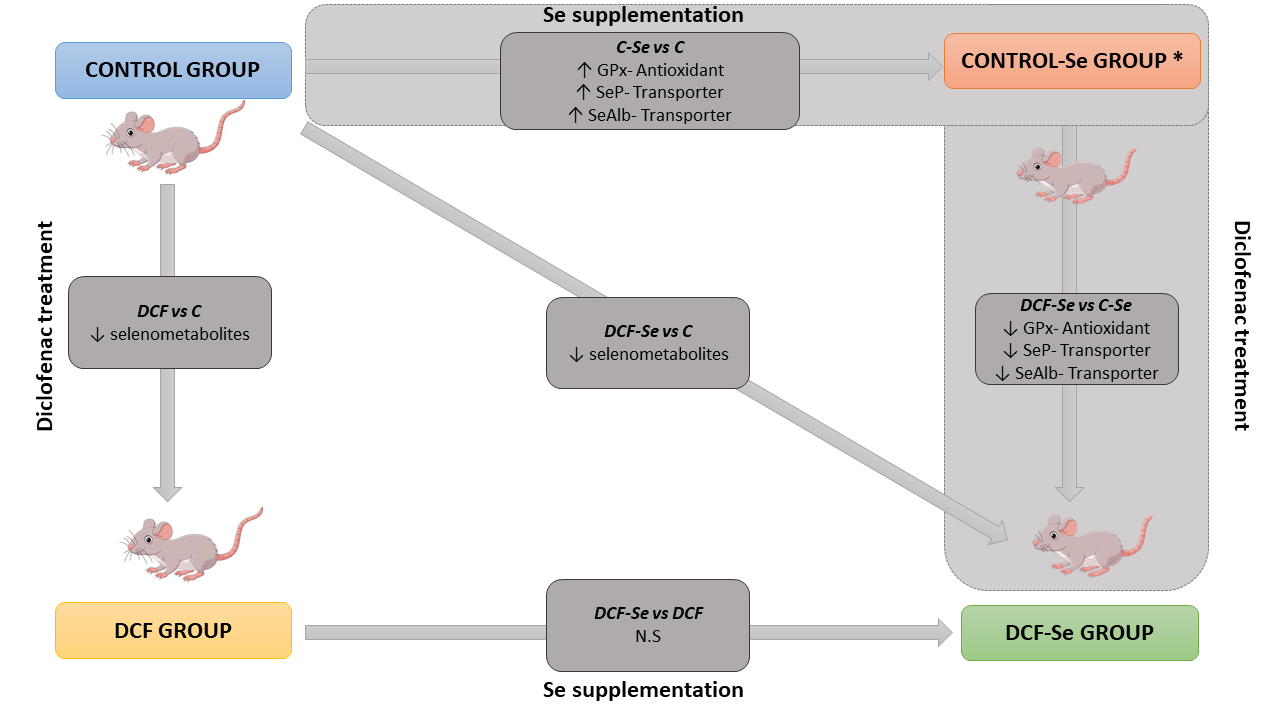


**Figure S2.** Score plot obtained by principal component analysis of gut extracts of *Mus muscul*us mice after GC-MS and UPLC-QTOF(+/-)-MS analysis. Color of tetrahedron represents each group: black (C); Blue (DCF); green (DCF-Se) and yellow (QCs).

**
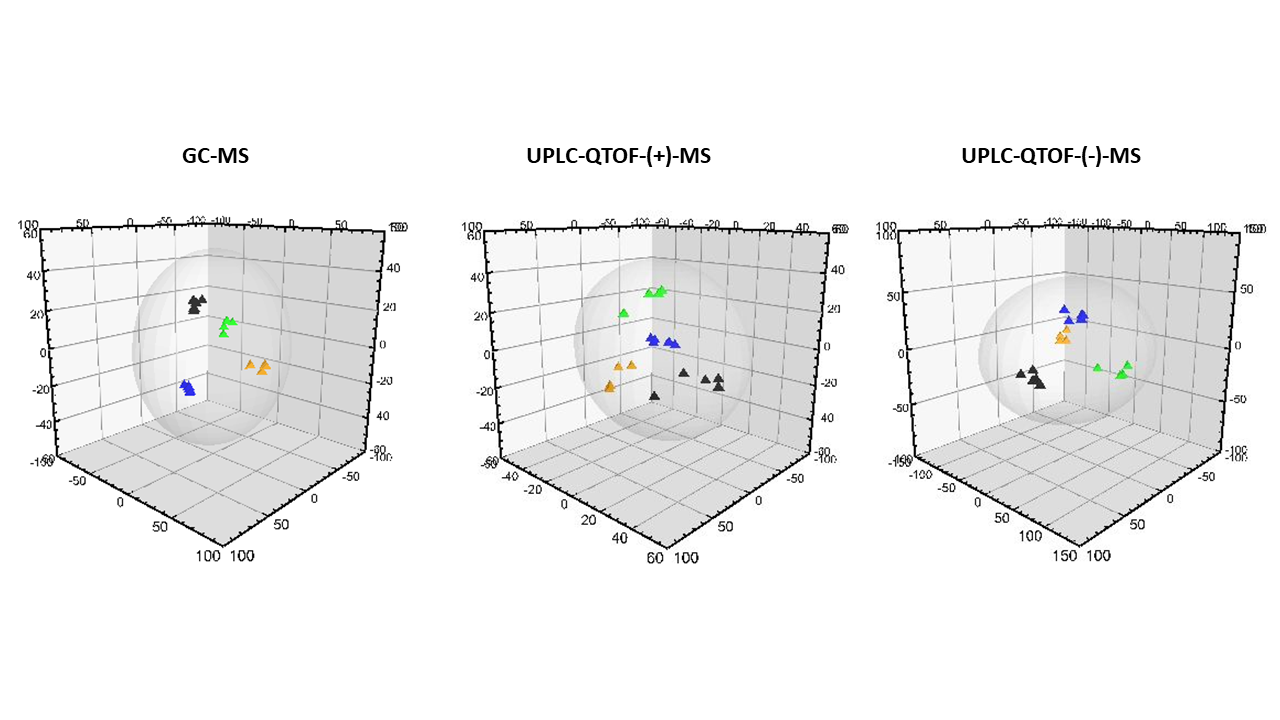
**

**Figure S3.** Score plot obtained by partial square discriminant analysis of gut extracts of *Mus muscul*us mice after GC-MS and UPLC-QTOF(+/-)-MS analysis. Color of tetrahedron represents each group: black (C); Blue (DCF) and green (DCF-Se).

**
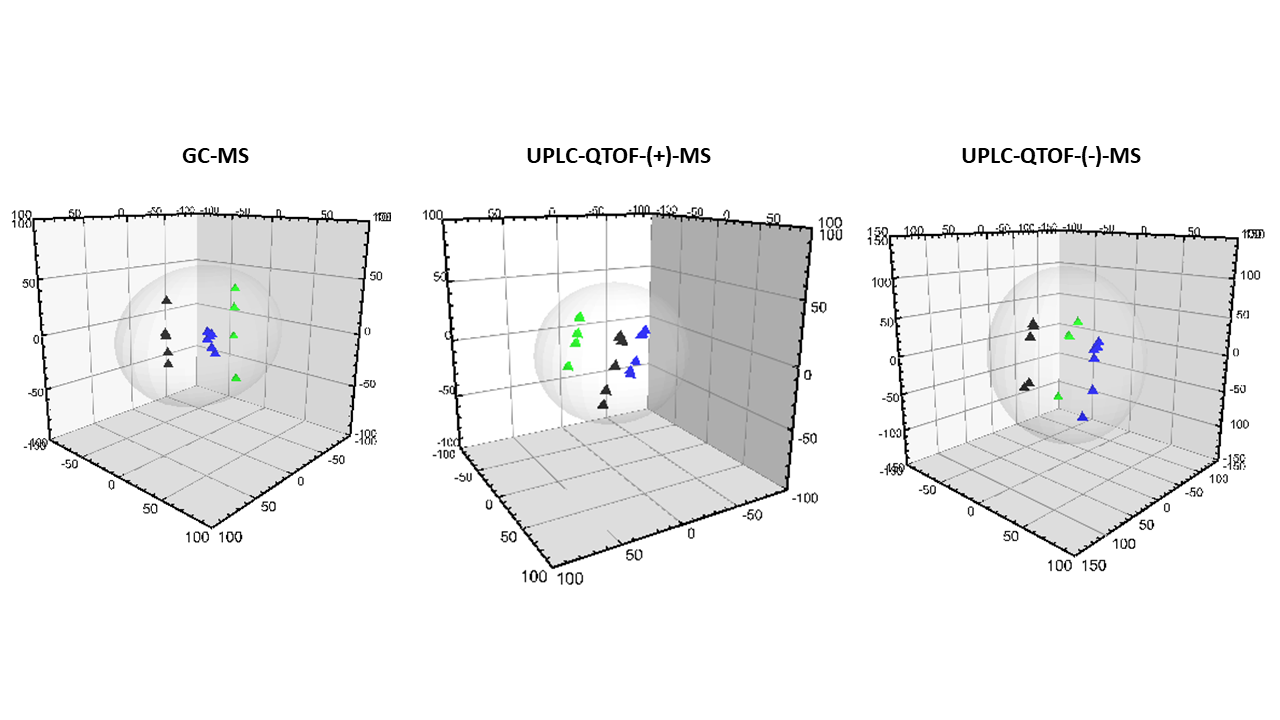
**

**Figure S4.** Score plots of 3D-PLS-DA of pairwise comparisons of gut samples of *Mus muscul*us mice after GC-MS and UPLC-QTOF(+/-)-MS analysis. Color of tetrahedron represents each group: black (C); Blue (DCF) and green (DCF-Se).

**
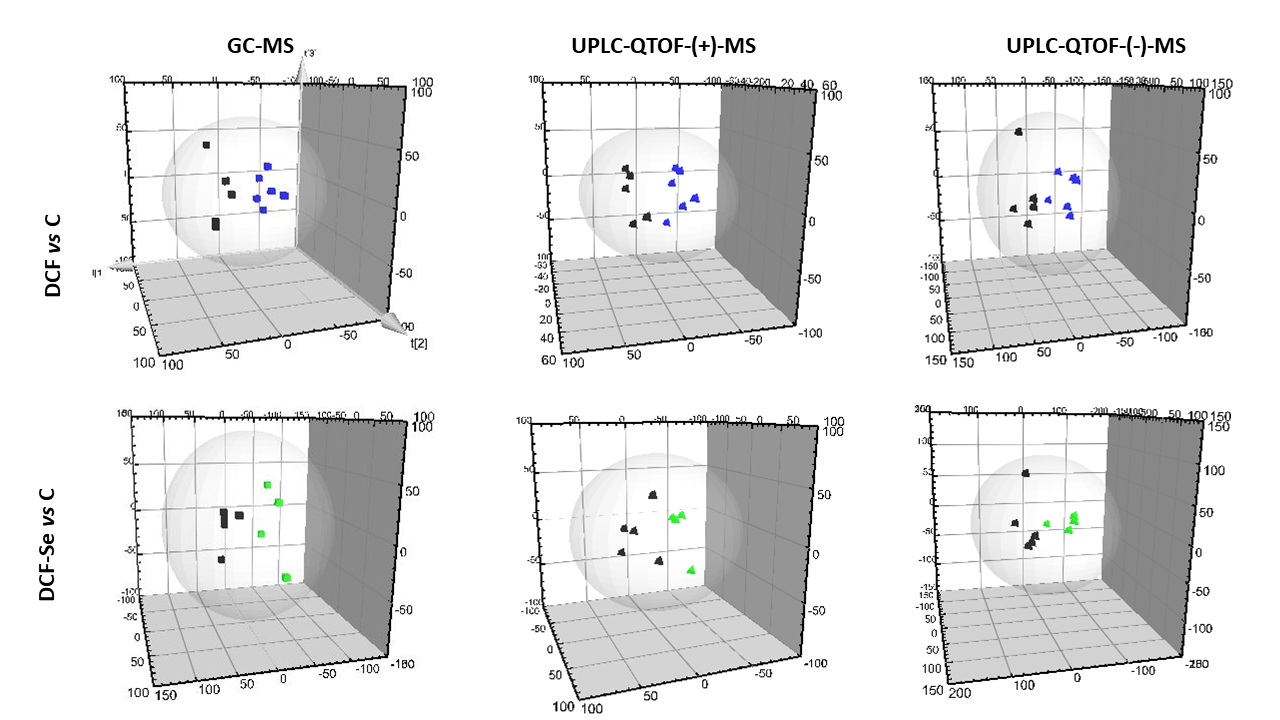
**

**Figure S5**. Venn diagram showing the number of metabolites identified after DCF exposure and Se supplementation.


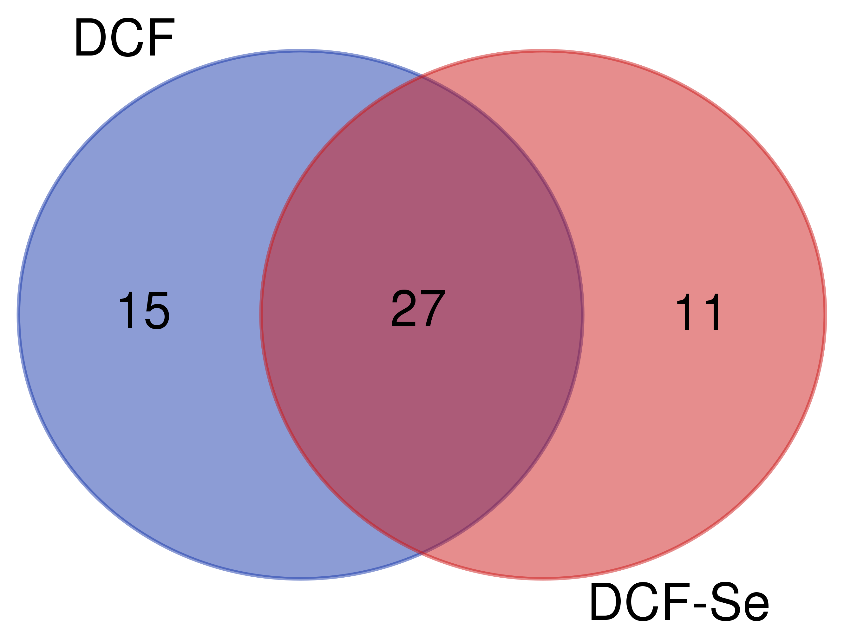


**Figure S6.** Class of altered metabolites after (A) DCF exposure and (B) DCF and Se supplementation in *Mus musculus* mice (inbred BALB/c).


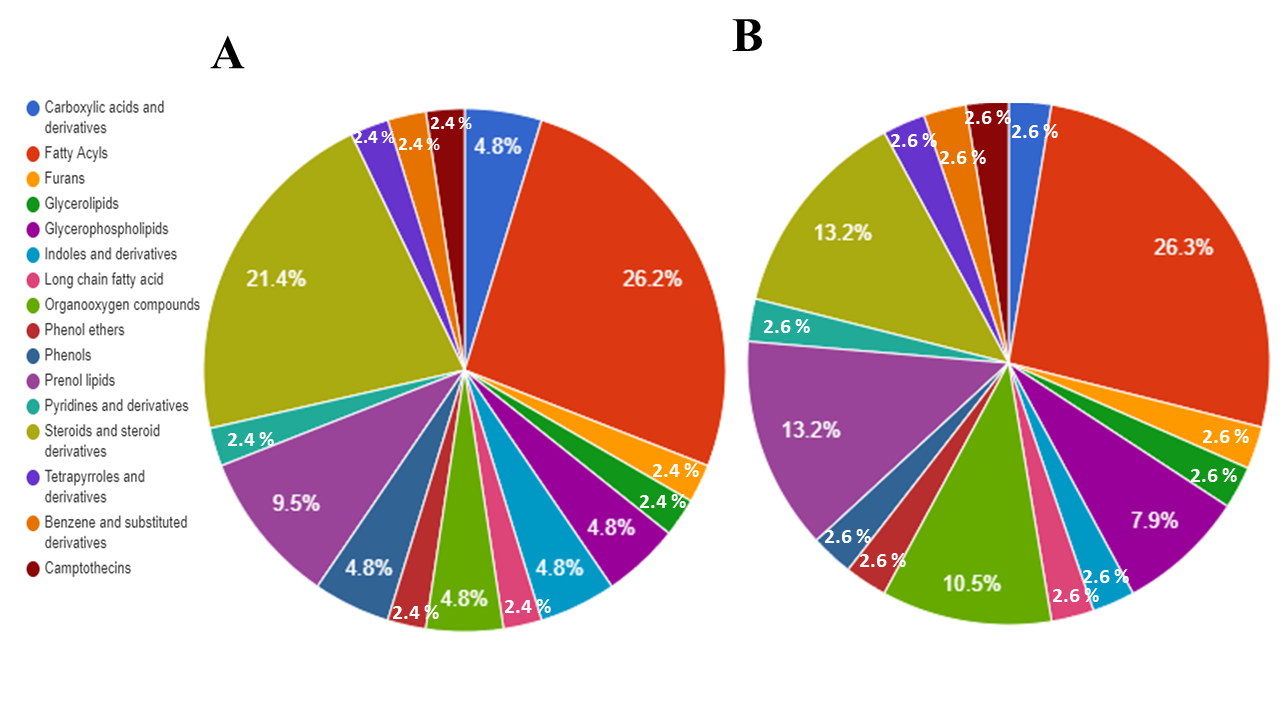


**Table S1**. R^2^ and Q^2^ parameters observed in partial least square discriminant analysis (PLS-DA) to determine the quality of the built models.

| **Metabolic platform** | **DCF vs C** | | **DCF-Se vs C** | |
| --- | --- | --- | --- | --- |
|  | **R^2^** | **Q^2^** | **R^2^** | **Q^2^** |
| GC-MS | 1 | 0.818 | 1 | 0.924 |
| LC-ESI(+)-MS | 1 | 0.833 | 1 | 0.879 |
| LC-ESI(-)-MS | 1 | 0.923 | 1 | 0.917 |

**Table S2.** Kovat’s Retention Index and targeted ions of identified metabolites by GC-MS.

| **Compound name** | **Retention Index** | **Theorical Mass (Da)** | **MW-derivatives (Da)** | **Derivatives** | **Targeted ions** |
| --- | --- | --- | --- | --- | --- |
| **Glycerol** | 1263 | 92.0473 | 308 | 3-TMS | 218/205/147 |
| **Linoleic acid** | 2135 | 280.4455 | 352 | 1-TMS | 337/262/178 |
| **Myo-Inositol** | 2280 | 180.0633 | 764 | 7-TMS | 318/299/191 |
| **Stearic acid** | 2319 | 284.2715 | 356 | 1-TMS | 341/257/145 |
| **Campesterol** | 3166 | 400.3705 | 472 | 1-TMS | 382, 367, 472 |
| **Sitosterol** | 3198 | 414.3861 | 486 | 1-TMS | 396/357/255 |

**Table S3.** Metabolites altered, ordered by class, in gut microbiota of *Mus musculus* mice after diclofenac exposure and Se supplementation obtained by UPLC-ESI(+/-)-QTOF-MS and GC-MS. RT: retention time, FC: Fold change, -: Not significant, lysophosphatidylcholine (LPC), lysophosphatidylethanolamine (LPE), Phosphatidylinositol (PI); Hydroxyeicosatetraenoic acids (HETE); Prostaglandin B1 (PGB1); monoglyceride (MG); *p*-value obtained from one way ANOVA followed by Tuckey Test and corrected by Benjamini-Hochberg multiple post-correction.

| **Compound** | **Mass** | **Retention Time (min)** | **Analytical platform** | **DCF vs C** | | **DCF-Se vs C** | | **DCF-Se vs DCF** | |
| --- | --- | --- | --- | --- | --- | --- | --- | --- | --- |
|  |  |  |  | **FC** | **p value** | **FC** | **p value** | **FC** | **p value** |
| **Benzene and substituted derivatives** |  |  |  |  |  |  |  |  | |
| Dodecylbenzene | 246.2347 | 15.03 | ESI+ | 0.59 | 0.0121 | - | - | - | - |
| **Benzoxazoles** | | | | | | | | | |
| 2-Methyl-4,5-benzoxazole | 133.0527 | 7.77 | ESI- | - | - | 1.69 | 0.0012 | 1.73 | 0.0051 |
| **Camptothecins** | | | | | | | | | |
| Topotecan | 421.1637 | 16.77 | ESI+ | 1.11 | 0.0325 | - | - | - | - |
| **Carboxylic acids and derivatives** | | | | | | | | | |
| 4-Amino-2-methylenebutanoic acid | 115.0633 | 2.13 | ESI+ | 0.81 | 0.0086 | - | - | - | - |
| n1,n8-diacetylspermidine | 229.179 | 17.04 | ESI+ | 1.21 | 0.0012 | 10.19 | 0.0116 | 8.41 | 0.0309 |
| **Fatty Acyls** | | | | | | | | | |
| Linoleic Acid | 280.4455 | 21.25 | GCMS | - | - | 2.51 | 0.0240 | - | - |
| Stearic Acid | 284.4772 | 25.62 | GCMS | 0.38 | 0.0280 | - | - | - | - |
| 15-HETE | 320.2351 | 6.96 | ESI- | - | - | 0.09 | 0.0004 | 1.30 | 0.0001 |
| 15-keto-PGE2 | 350.2093 | 7.74 | ESI- | - | - | 0.01 | 0.0006 | 0.01 | 0.0004 |
| Dodecanedioic acid | 230.1518 | 7.03 | ESI- | 0.40 | 0.0233 | 0.59 | 0.0001 | 1.46 | 0.0002 |
| Nonate | 188.1048 | 8.54 | ESI- | 1.20 | 0.0001 | 9.50 | 0.0001 | 7.89 | 0.0002 |
| Tetradecanedioic acid | 258.1831 | 7.01 | ESI- | 1.46 | 0.0001 | - | - | 3.33 | 0.0415 |
| trans-9, trans-11-octadecadienoic acid | 280.2402 | 14.78 | ESI+ | 0.05 | 0.0004 | 2.01 | 0.0480 | 44.25 | 0.0335 |
| Traumatic Acid | 228.1361 | 9.35 | ESI- | 1.64 | 0.0474 | - | - | - | - |
| 16-Oxo-palmitate | 269.2122 | 8.01 | ESI- | 0.38 | 0.0234 | 0.38 | 0.0184 | - | - |
| 3-oxo-tetradecanoic acid | 242.1881 | 8.62 | ESI- | 0.62 | 0.0005 | - | - | 1.73 | 0.0001 |
| 6-Hydroxypentadecanedioic acid | 288.1936 | 2.34 | ESI- | - | - | 0.64 | 0.0175 | - | - |
| Butyl butyryllactate | 216.1361 | 4.27 | ESI- | 0.59 | 0.0074 | 0.36 | 0.0169 | 0.61 | 0.0077 |
| PGB1 | 336.2300 | 9.46 | ESI- | 1.71 | 0.0213 | 5.74 | 0.0001 | 3.35 | 0.0001 |
| Suberic acid | 174.0892 | 2.02 | ESI- | 0.78 | 0.0026 | - | - | 1.33 | 0.0462 |
| **Furans** | | | | | | | | | |
| Methyl 2-furoate | 126.0316 | 14.45 | ESI+ | 0.13 | 0.0335 | 0.08 | 0.0194 | 0.61 | 0.0497 |
| **Glycerolipids** | | | | | | | | | |
| MG(14:1) | 300.2300 | 9.94 | ESI- | 0.64 | 0.0001 | 0.60 | 0.0001 | - | - |
| **Glycerophospholipids** | | | | | | | | | |
| LysoPC(22:1) | 577.4107 | 10.94 | ESI+ | - | - | 1.89 | 0.0030 | - | - |
| LysoPE(14:0) | 425.2542 | 9.37 | ESI- | 0.37 | 0.0036 | 0.46 | 0.0011 | 1.24 | 0.0049 |
| PI(18:0/16:0) | 719.5465 | 6.97 | ESI- | 1.73 | 0.0012 | 1.98 | 0.0045 | 1.15 | 0.0027 |
| **Hydroxy acids and derivatives** | | | | | | | | | |
| 12-Hydroxydodecanoic acid | 216.1725 | 8.78 | ESI- | - | - | 3.02 | 0.0006 | 3.01 | 0.0009 |
| **Indoles and derivatives** | | | | | | | | | |
| 5-Methoxytryptophan | 234.1004 | 5.75 | ESI+ | 0.79 | 0.0019 | - | - | - | - |
| xi-2,3-Dihydro-2-oxo-1H-indole-3-acetic acid | 191.0582 | 17.85 | ESI+ | 1.78 | 0.0012 | 2.00 | 0.0393 | - | - |
| **Long chain fatty acid** | | | | | | | | | |
| 2-methoxy-hexadecanoic acid | 286.4 | 10.59 | ESI- | 0.60 | 0.0057 | 2.95 | 0.0001 | 4.90 | 0.0006 |
| **Organooxygen compounds** | | | | | | | | | |
| Glycerol | 92.0473 | 10.55 | GCMS | - | - | 3.45 | 0.0163 | - | - |
| Myo-inositol | 180.1559 | 22.80 | GCMS | - | - | 12.55 | 0.0300 | 21.78 | 0.0190 |
| DHAP(18:0) | 436.2589 | 7.73 | ESI- | 2.55 | 0.0002 | 3.74 | 0.0011 | 1.47 | 0.0235 |
| 11,13-Hexacosanedione | 394.381 | 15.13 | ESI+ | 0.53 | 0.0014 | 0.33 | 0.0499 | 0.62 | 0.0030 |
| **Phenol ethers** | | | | | | | | | |
| alpha,alpha-Dimethylanisalacetone | 204.115 | 15.93 | ESI+ | 2.54 | 0.0039 | 2.32 | 0.0002 | - | - |
| **Phenols** | | | | | | | | | |
| 3-Pentadecylphenol | 304.2766 | 10.89 | ESI- | 0.32 | 0.0147 | 0.11 | 0.0005 | 0.36 | 0.0282 |
| Dihydrocapsaicin | 307.2147 | 16.10 | ESI+ | 3.61 | 0.0026 | - | - | - | - |
| **Prenol lipids** | | | | | | | | | |
| 7(14)-Bisabolene-2,3,10,11-tetrol | 272.1987 | 8.80 | ESI- | 0.84 | 0.0172 | 1.67 | 0.0264 | 1.99 | 0.0030 |
| Cuminaldehyde | 148.0888 | 14.72 | ESI+ | 1.23 | 0.0229 | 0.35 | 0.0020 | 0.28 | 0.0082 |
| Linalyl propionate | 210.1619 | 4.83 | ESI+ | 0.39 | 0.0152 | - | - | - | - |
| Menthyl acetoacetate | 240.1725 | 3.14 | ESI- | - | - | 0.87 | 0.0001 | - | - |
| Monomenthyl succinate | 256.1674 | 9.50 | ESI- | 2.51 | 0.0279 | 0.43 | 0.0067 | 0.17 | 0.0195 |
| Ubiquinone (Q2) | 318.1831 | 8.15 | ESI- | - | - | 1.31 | 0.0120 | 1.21 | 0.0002 |
| **Pyridines and derivatives** | | | | | | | | | |
| 2-Hydroxynicotinic acid | 139.0269 | 13.16 | ESI+ | 0.24 | 0.0274 | 0.51 | 0.0459 | - | - |
| **Steroids and steroid derivatives** | | | | | | | | | |
| Sitosterol | 414.3861 | 30.50 | GCMS | 1.21 | 0.0390 | - | - | 0.12 | 0.0354 |
| Campesterol | 400.3705 | 29.58 | GC-MS | 1.54 | 0.0019 | 1.12 | 0.0346 | - | - |
| 3alpha,7alpha-Dihydroxy-5beta-cholestan-26-oic acid | 434.3396 | 10.86 | ESI- | 0.38 | 0.0378 | 0.34 | 0.0015 | 0.89 | 0.0419 |
| Cholesterol sulfate | 466.3116 | 9.87 | ESI- | 0.32 | 0.0011 | - | - | - | - |
| Cholic acid | 408.2875 | 7.09 | ESI- | 4.98 | 0.0225 | 0.48 | 0.0048 | 0.10 | 0.0003 |
| Tetrahydrocorticosterone | 350.2457 | 9.98 | ESI- | 0.87 | 0.0169 | 0.39 | 0.0117 | 0.45 | 0.0309 |
| (3beta,5alpha,24S)-Stigmasta-7,25-dien-3-ol | 412.3705 | 5.38 | ESI+ | 2.01 | 0.0054 | - | - | - | - |
| 3-epi-6-Deoxocathasterone | 418.3810 | 9.95 | ESI- | 1.60 | 0.0106 | - | - | 0.62 | 0.0001 |
| Chenodeoxycholic Acid | 392.2926 | 10.78 | ESI- | 0.50 | 0.0220 | 0.14 | 0.0151 | 0.28 | 0.0099 |
| **Tetrapyrroles and derivatives** | | | | | | | | | |
| Deuteroporphyrin IX | 510.2267 | 13.17 | ESI+ | 0.24 | 0.0287 | 0.56 | 0.0346 | - | - |

**Table S4.** List of common and different altered metabolites for DCF group and DCF plus Se group.

| **Exclusive metabolites DCF group** | **Common metabolites** | **Exclusive metabolites DCF-Se group** |
| --- | --- | --- |
| Cholesterol sulfate | 7(14)-Bisabolene-2,3,10,11-tetrol | Menthyl acetoacetate |
| Dodecylbenzene | alpha,alpha-Dimethylanisalacetone | 2-Methyl-4,5-benzoxazole |
| Linalyl propionate | 2-methoxy-hexadecanoic acid | Linoleic Acid |
| 3-oxo-tetradecanoic acid | Cholic acid | LysoPC(22:1) |
| Sitosterol | Monomenthyl succinate | 15-keto-PGE2 |
| Dihydrocapsaicin | 11,13-Hexacosanedione | Ubiquinone (Q2) |
| Topotecan | PI(18:0/16:0) | Myo-inositol |
| 4-Amino-2-methylenebutanoic acid | 3alpha,7alpha-Dihydroxy-5beta-cholestan-26-oic acid | Glycerol |
| Stearic Acid | Nonate | 15-HETE |
| 5-Methoxytryptophan | Cuminaldehyde | 12-Hydroxydodecanoic acid |
| Suberic acid | Methyl 2-furoate | 6-Hydroxypentadecanedioic acid |
| Traumatic Acid | LysoPE(14:0) |  |
| Tetradecanedioic acid | 2-Hydroxynicotinic acid |  |
| (3beta,5alpha,24S)-Stigmasta-7,25-dien-3-ol | Deuteroporphyrin IX |  |
| 3-epi-6-Deoxocathasterone | DHAP(18:0) |  |
|  | 16-Oxo-palmitate |  |
|  | xi-2,3-Dihydro-2-oxo-1H-indole-3-acetic acid |  |
|  | Dodecanedioic acid |  |
|  | Chenodeoxycholic Acid |  |
|  | MG(14:1) |  |
|  | Campesterol |  |
|  | 3-Pentadecylphenol |  |
|  | n1,n8-diacetylspermidine |  |
|  | Butyl butyryllactate |  |
|  | PGB1 |  |
|  | Tetrahydrocorticosterone |  |
|  | trans-9, trans-11-octadecadienoic acid |  |

**Table S5.** Results of match status, which is the number of metabolites involved in the pathway, p-value and pathway impact scores for the significant pathways after DCF and Se exposure.

| **Pathway** | **Pathway Name** | **Match status** | **p-value** | **Impact** |
| --- | --- | --- | --- | --- |
| **DCF** | [Primary](https://www.metaboanalyst.ca/faces/Secure/pathway/ResultView.xhtml) bile biosysnthesis | 3 | 0.018 | 0.032 |
| **DCF-Se** | [[Primary](https://www.metaboanalyst.ca/faces/Secure/pathway/ResultView.xhtml) bile biosysnthesis](https://www.metaboanalyst.ca/faces/Secure/pathway/ResultView.xhtml) | 3 | 0.015 | 0.032 |
|  | [Galactose metabolism](https://www.metaboanalyst.ca/faces/Secure/pathway/ResultView.xhtml) | 2 | 0.039 | 0 |
|  | [Linoleic acid metabolism](https://www.metaboanalyst.ca/faces/Secure/pathway/ResultView.xhtml) | 1 | 0.048 | 1 |

**Table S6.** Correlation analysis between gut metabolites and gut microbes in mice exposed to DCF.

| **Bacteria** | **Metabolite** | **Spearman** | **p-level** |
| --- | --- | --- | --- |
| *Candidatus_Saccharimonas* | Cholic acid | -0.9429 | 0.0048 |
|  | Dodecanedioic acid | 0.9276 | 0.0077 |
| *Pseudomonas* | xi-2,3-Dihydro-2-oxo-1H-indole-3-acetic acid | -0.8857 | 0.0188 |
|  | Dodecanedioic acid | 0.8117 | 0.0499 |
|  | Cholic acid | -0.8286 | 0.0416 |
| *Acinetobacter* | 12-Hydroxydodecanoic acid | 0.9411 | 0.0051 |
|  | Dodecanedioic acid | 0.8933 | 0.0165 |
|  | Cuminaldehyde | -0.8804 | 0.0206 |
|  | Cholic acid | -0.8804 | 0.0206 |
|  | MG(14:1) | -0.8804 | 0.0206 |
| *[Eubacterium]_xylanophilum_group* | MG(14:1) | -0.9429 | 0.0048 |
|  | 12-Hydroxydodecanoic acid | 0.8857 | 0.0188 |
| *Bacteroides* | 11,13-Hexacosanedione | -0.8857 | 0.0188 |
|  | Tetradecanedioic acid | 0.8857 | 0.0188 |
| *[Eubacterium]_coprostanoligenes_group* | 11,13-Hexacosanedione | -0.8857 | 0.0188 |
|  | Tetradecanedioic acid | 0.8857 | 0.0188 |
| *Pediococcus* | 11,13-Hexacosanedione | -0.9411 | 0.0051 |
|  | 2-Methyl-4,5-benzoxazole | 0.8804 | 0.0206 |
|  | Cuminaldehyde | -0.8197 | 0.0458 |
| *Lactobacillus* | 2-Methyl-4,5-benzoxazole | 0.8857 | 0.0188 |
|  | 11,13-Hexacosanedione | -0.8286 | 0.0416 |
| *Muribaculaceae* | 2-Methyl-4,5-benzoxazole | 0.9429 | 0.0048 |
| *Lachnospiraceae_UCG-008* | 2-Methyl-4,5-benzoxazole | 0.9429 | 0.0048 |
| *Marvinbryantia* | PI(18:0/16:0) | 0.9429 | 0.0048 |
|  | Chenodeoxycholic Acid | -0.8857 | 0.0188 |
|  | LysoPE(0:0/14:0) | -0.8286 | 0.0416 |
|  | Traumatic Acid | 0.8286 | 0.0416 |
| *Odoribacter* | 3-epi-6-Deoxocathasterone | -0.8857 | 0.0188 |
|  | PI(18:0/16:0) | 0.8857 | 0.0188 |
|  | Cholesterol sulfate | 0.8407 | 0.0361 |
| *Roseburia* | Tetradecanedioic acid | 0.8857 | 0.0188 |
|  | L-Menthyl acetoacetate | -0.8407 | 0.0361 |
|  | Methyl 2-furoate | -0.8286 | 0.0416 |
|  | Ubiquinone (Q2) | 0.8286 | 0.0416 |
|  | 3-oxo-tetradecanoic acid | 0.8286 | 0.0416 |
| *Alloprevotella* | 3-epi-6-Deoxocathasterone | -0.8857 | 0.0188 |
|  | L-Menthyl acetoacetate | -0.8117 | 0.0499 |
| *[Eubacterium]_siraeum_group* | Cholesterol sulfate | -0.9276 | 0.0077 |
|  | Monomenthyl succinate | -0.8986 | 0.0149 |
|  | PGB1 | 0.8286 | 0.0416 |
| *Ruminococcus* | 3-epi-6-Deoxocathasterone | 0.9429 | 0.0048 |
| *Desulfovibrio* | PI(18:0/16:0) | -0.8286 | 0.0416 |
|  | Traumatic Acid | -0.8286 | 0.0416 |
|  | Cholesterol sulfate | -0.8117 | 0.0499 |
| *Enterorhabdus* | PI(18:0/16:0) | -0.9429 | 0.0048 |
|  | Topotecan | 0.8857 | 0.0188 |
|  | LysoPE(14:0) | 0.8286 | 0.0416 |
| *Olsenella* | Monomenthyl succinate | -0.8986 | 0.0149 |
|  | Cholic acid | 0.8857 | 0.0188 |
|  | Chenodeoxycholic Acid | -0.7286 | 0.0416 |
| *Lachnospiraceae_UCG-006* | Cholic acid | 0.9429 | 0.0048 |
| *Blautia* | L-Menthyl acetoacetate | 0.9276 | 0.0077 |
|  | 7(14)-Bisabolene-2,3,10,11-tetrol | -0.8857 | 0.0188 |
|  | Ubiquinone (Q2) | -0.8286 | 0.0416 |
| *Ileibacterium* | 7(14)-Bisabolene-2,3,10,11-tetrol | -0.9429 | 0.0048 |
| *Oscillibacter* | 4-Amino-2-methylenebutanoic acid | -0.8407 | 0.0361 |
|  | 15-keto-PGE2 | -0.8286 | 0.0416 |
| *Bilophila* | 4-Amino-2-methylenebutanoic acid | -0.8986 | 0.0149 |
|  | Linalyl propionate | -0.8286 | 0.0416 |
|  | 7(14)-Bisabolene-2,3,10,11-tetrol | -0.8286 | 0.0416 |
| *Muribaculum* | Butyl butyryllactate | 0.8986 | 0.0149 |
| *GCA-900066575* | 15-keto-PGE2 | -0.9429 | 0.0048 |
|  | Nonate | -0.8117 | 0.0499 |
| *Clostridia_vadinBB60_group* | Chenodeoxycholic Acid | -0.8857 | 0.0188 |
|  | Tetradecanedioic acid | 0.8286 | 0.0416 |
|  | 16-Oxo-palmitate | -0.8117 | 0.0499 |
| *Lachnospiraceae_UCG-001* | Tetrahydrocorticosterone | 0.8857 | 0.0188 |
|  | 3-Pentadecylphenol | 0.8407 | 0.0361 |
|  | Nonate | -0.8117 | 0.0499 |
| *Akkermansia* | Tetradecanedioic acid | 0.8286 | 0.0416 |
|  | Tetrahydrocorticosterone | 0.8286 | 0.0416 |
| *Parasutterella* | MG(14:1) | 0.8286 | 0.0416 |
| *Dubosiella* | MG(14:1) | 0.8286 | 0.0416 |
| *RF39* | PI(18:0/16:0) | 0.8286 | 0.0416 |
|  | Traumatic Acid | 0.8286 | 0.0416 |
|  | Cholesterol sulfate | 0.8117 | 0.0499 |
| *Clostridia_UCG-014* | PI(18:0/16:0) | 0.8857 | 0.0188 |
|  | Cholesterol sulfate | 0.8117 | 0.0499 |
| *UCG-005* | Traumatic Acid | 0.8857 | 0.0188 |
| *Lachnospiraceae_NK4A136_group* | n1,n8-diacetylspermidine | 0.8857 | 0.0188 |
|  | 12-Hydroxydodecanoic acid | -0.8286 | 0.0416 |
| *Alistipes* | PI(18:0/16:0) | 0.8286 | 0.0416 |
| *Helicobacter* | Tetrahydrocorticosterone | 0.8857 | 0.0188 |
| *NK4A214_group* | n1,n8-diacetylspermidine | 0.8117 | 0.0499 |
| *ASF356* | n1,n8-diacetylspermidine | 0.8857 | 0.0188 |
| *Prevotellaceae_UCG-001* | 2-Methyl-4,5-benzoxazole | -0.8286 | 0.0416 |
| *UCG-010* | Suberic acid | 0.8857 | 0.0188 |
|  | Tetradecanedioic acid | -0.8286 | 0.0416 |
|  | 3-oxo-tetradecanoic acid | -0.8286 | 0.0416 |
| *Butyricicoccus* | 11,13-Hexacosanedione | 0.9276 | 0.0077 |
|  | PGB1 | -0.8407 | 0.0361 |
|  | Tetradecanedioic acid | -0.8117 | 0.0499 |
|  | 2-Methyl-4,5-benzoxazole | -0.8117 | 0.0499 |
| *Lachnoclostridium* | 3-Pentadecylphenol | -0.8986 | 0.0149 |
|  | Nonate | 0.8407 | 0.0361 |
|  | xi-2,3-Dihydro-2-oxo-1H-indole-3-acetic acid | 0.8857 | 0.0188 |
|  | Dodecylbenzene | 0.8286 | 0.0416 |
|  | Dihydrocapsaicin | 0.8286 | 0.0416 |
| *Faecalibaculum* | Dodecylbenzene | 0.9276 | 0.0077 |
|  | Dodecanedioic acid | -0.8824 | 0.0199 |
|  | 12-Hydroxydodecanoic acid | -0.8407 | 0.0361 |
|  | 11,13-Hexacosanedione | 0.8117 | 0.0499 |
| *Colidextribacter* | Dodecanedioic acid | -0.9276 | 0.0077 |
|  | 11,13-Hexacosanedione | 0.8286 | 0.0416 |
| *Rikenellaceae_RC9_gut_group* | Tetradecanedioic acid | -0.8286 | 0.0416 |
|  | 15-keto-PGE2 | 0.8286 | 0.0416 |
|  | Tetrahydrocorticosterone | -0.8286 | 0.0416 |
|  | 3-oxo-tetradecanoic acid | -0.8286 | 0.0416 |
| *Anaerotruncus* | Tetrahydrocorticosterone | -0.8286 | 0.0416 |
|  | 3-Pentadecylphenol | -0.8117 | 0.0499 |
| *Turicibacter* | Tetrahydrocorticosterone | -0.9276 | 0.0077 |
|  | 15-keto-PGE2 | 0.8407 | 0.0361 |
| *Parabacteroides* | 3-Pentadecylphenol | -0.9276 | 0.0077 |
|  | Butyl butyryllactate | 0.8857 | 0.0188 |
| *Sphingobacterium* | 15-keto-PGE2 | 0.9429 | 0.0048 |
| *Mucispirillum* | Nonate | 0.8986 | 0.0149 |
|  | 15-keto-PGE2 | 0.8286 | 0.0416 |
| *A2* | Topotecan | 0.8857 | 0.0188 |
|  | Linalyl propionate | 0.8857 | 0.0188 |
|  | 15-keto-PGE2 | 0.8857 | 0.0188 |
